# Generation of Diverse Biological Forms through Combinatorial Interactions between Tissue Polarity and Growth

**DOI:** 10.1371/journal.pcbi.1002071

**Published:** 2011-06-16

**Authors:** Richard Kennaway, Enrico Coen, Amelia Green, Andrew Bangham

**Affiliations:** 1 School of Computing Sciences, University of East Anglia, Norwich, United Kingdom; 2 John Innes Centre, Norwich, United Kingdom; University of California San Diego, United States of America

## Abstract

A major problem in biology is to understand how complex tissue shapes may arise through growth. In many cases this process involves preferential growth along particular orientations raising the question of how these orientations are specified. One view is that orientations are specified through stresses in the tissue (axiality-based system). Another possibility is that orientations can be specified independently of stresses through molecular signalling (polarity-based system). The axiality-based system has recently been explored through computational modelling. Here we develop and apply a polarity-based system which we call the Growing Polarised Tissue (GPT) framework. Tissue is treated as a continuous material within which regionally expressed factors under genetic control may interact and propagate. Polarity is established by signals that propagate through the tissue and is anchored in regions termed tissue polarity organisers that are also under genetic control. Rates of growth parallel or perpendicular to the local polarity may then be specified through a regulatory network. The resulting growth depends on how specified growth patterns interact within the constraints of mechanically connected tissue. This constraint leads to the emergence of features such as curvature that were not directly specified by the regulatory networks. Resultant growth feeds back to influence spatial arrangements and local orientations of tissue, allowing complex shapes to emerge from simple rules. Moreover, asymmetries may emerge through interactions between polarity fields. We illustrate the value of the GPT-framework for understanding morphogenesis by applying it to a growing Snapdragon flower and indicate how the underlying hypotheses may be tested by computational simulation. We propose that combinatorial intractions between orientations and rates of growth, which are a key feature of polarity-based systems, have been exploited during evolution to generate a range of observed biological shapes.

## Author Summary

How do genes control the growth of cells into complex tissue shapes such as flowers, wings or hearts? A key requirement is that genes must be able to modulate growth along particular directions. Two mechanisms have been proposed for how this may work; one based on the directions of mechanical stresses in the tissue and the other on molecular signals that propagate and provide local polarities. Here we show how a polarity-based system has the advantage of being able to act in combination with growth rates to generate a wide range of shapes. By applying this system to the development of the Snapdragon flower, we show, by comparison of computational simulations with actual flower development, how a simple set of polarity controls may underlie the formation of complex biological structures.

## Introduction

Although there have been many experimental and theoretical studies on patterns of gene activities and their establishment in animals and plants [1]–[6] much less is known about how patterns of activity are linked to tissue growth and deformation. Addressing this problem represents a challenge because final form is usually not a direct readout of locally specified properties, but depends on mechanical constraints from neighbouring regions. For example, if the margin of a leaf has a higher specified growth rate than the centre, a wavy edge will emerge. The wavy edge is not directly specified but is a feature that emerges through the interaction between patterns of specified growth and the mechanical constraints of tissue continuity [7]. In such cases we may distinguish between *specified growth*, which is the growth that would be attained if each region grew independently of its neighbours (i.e. in mechanical isolation), and *resultant growth*, which is the growth observed when mechanical constraints of neighbours are taken into account (i.e. mechanically connected tissue). In animal systems a similar distinction is made between an imposed active deformation, and an elastic passive deformation [8].

Resultant growth can be measured experimentally by tracking tissue deformations over time [9]–[13]. However, to understand the mechanisms by which resultant growth arises we need to know how genes influence specified growth. Where specified growth is isotropic, genes need to control a single parameter, the local rate of growth. However, in many cases specified growth may be anisotropic requiring orientations as well as rates of growth to be under genetic control. Controlling orientations of growth requires a local axis to be defined (i.e. axiality, represented as a field of lines). In this respect growth is similar to stress which also has axiality. This similarity has led to the suggestion that stresses provide the primary cues for orienting growth. According to such a *stress-based axiality* mechanism, gene activity influences stresses in the tissue, the orientations of which are transduced to influence molecular properties of cells such as the cytoskeleton. These in turn modulate growth orientations which may further feed back to influence the pattern of stresses [14]–[18]. Recent support for such mechanisms in plants have come from studies of the effect of stresses on microtubule patterns [19].

A different way of specifying orientations of growth is through differential concentrations of signalling molecules. The varying concentrations define a local (cellular) polarity which includes both axiality and directional components (represented by a field of arrows). The axiality component is then used to orient growth. In this *polarity-based axiality* system, genes influence the distribution of signalling molecules which define a coordinated field of polarities. Incorporation of mechanical constraints then leads to resultant growth, which may feed back to influence, for example, tissue polarity orientations. In support of this system, there is considerable evidence that polarity is prevalent in biological tissues and may modulate growth [20]–[24]. For example, planar cell polarity (PCP) systems have been described in animals and implicated in processes such as growth of wings in Drosophila and convergent-extension in vertebrates [25]–[27]. Similarly, the polarised distribution of auxin transporters (PIN molecules) has been shown to be important for outgrowths of primordia in plants [28].

Elements of the stress-based axiality system have recently been modelled [29], [30]. Here we describe a framework and software implementation for the alternative polarity-based axiality approach, which we call GPT-framework. ur software, called GFtbox, is a MATLAB application available from http://www.uea.ac.uk/cmp/research/cmpbio/GFtbox. This framework was developed with plant growth in mind, although it may also be useful for modelling animal systems where cell movement is limited. In accompanying papers we show how a biologically relevant model can be derived using the GPT-framework [31], [32]. This model of Snapdragon flower development is constrained by a range of experimental data including gene expression patterns, mutant phenotypes, clonal analysis, growth dynamics and changes in geometry. It provides a working hypothesis for how growth is specified and shows how reorientation of growth can account for key observations. In this paper we explore a series of simplified models which illustrate how growth and polarity may interact combinatorially during morphogenesis to generate a wide range of forms. The results highlight the value of being able to specify orientation independently of stresses in the generation of complex tissue shapes. In addition, we provide the theoretical foundations on which our modelling depends.

## Results

### Overview of the GPT-framework

Modelling the genetic control of tissue growth requires the incorporation of gene regulatory networks and signal propogation within a growing, mechanically connected, tissue. In the GPT-framework, tissue is treated as a continuous sheet of material with two surfaces and a thickness, here termed the canvas. Biologically, the canvas may correspond to a sheet of cells, single cells or subcellular components (e.g. walls). Regulatory factors are distributed over the canvas and may interact and propagate, allowing particular patterns and local polarities to be specified. Regulatory factors can be classified into two types. *Identity factors* do not propagate within the canvas, while *signalling factors* can. The regulatory factors specify a growth tensor field which describes the specified rates of growth parallel and perpendicular to the local polarity. Elasticity theory is used to compute the resultant deformation of the canvas. This deformation modifies the relationships within the canvas and thus feeds back to influence the regulatory factors. Our implementation (*GFtbox*) is specialised towards tissues that grow as sheets, such as petals or leaves, but the basic concepts are also applicable to bulk three-dimensional and flat two-dimensional tissues.

In the results we study the interactions between tissue polarity and differential growth in the generation of shape through a series of models. For convenience each example has a setup phase during which the shape of the initial canvas and distribution of regional identities and signalling factors is established, and three components that form the model. (1) A Polariser Regulatory Network (PRN) controls the activity of various organisers from which tissue polarity information propagates. There are two types of organiser, termed 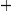
organiser and 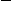
organiser. As a convention, we show polarity pointing away from 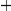
organisers, and towards 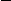
organisers. Polarity propagation is implemented through a signalling factor called POLARISER (POL), the gradient of which defines local polarity. The PRN controls production and degradation of POL at organisers that anchor the polarity. POL may also be produced and degraded at a background rate throughout the canvas. (2) A gene regulatory network (GRN) controls the activity of identity or signalling factors encoded by genes. (3) A growth rate regulatory network (KRN) determines how identity or signalling factors influence specified growth rates parallel to, 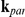, and perpendicular to, 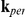, local polarity. The KRN also specifies the growth in thickness, 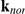. The specified growth rates for a region of the canvas are equivalent to the growth that would arise without the constraints of surrounding material (see Methods).

In the first time step the specified growth field is applied to the initial canvas which may then distort through mechanical interactions in the continuous material (modelled according to elasticity theory, see Methods). The result is a slight deformation of the canvas (resultant growth field) that takes the regions of identity factors with it. Where a region containing an identity factor expands, that region inherits the properties of the parent region, so maintaining boundaries. In such new volumes, the concentrations of signalling factors are interpolated from the parent surrounding regions and then further adjusted according to their production, dilution, propagation and decay rates. The deformed canvas and expression pattern provides the starting point for the next time step and the sequence is reiterated. To verify the computational correctness of *GFtbox*, results were computed for several situations where analytical solutions are possible (see Text S1). In the following we explore combinatorial interactions between polarity and growth through a series of simple cases. We first consider deformations in 2D.

### Interaction between growth and polarity (2D)

**Figure 1:**
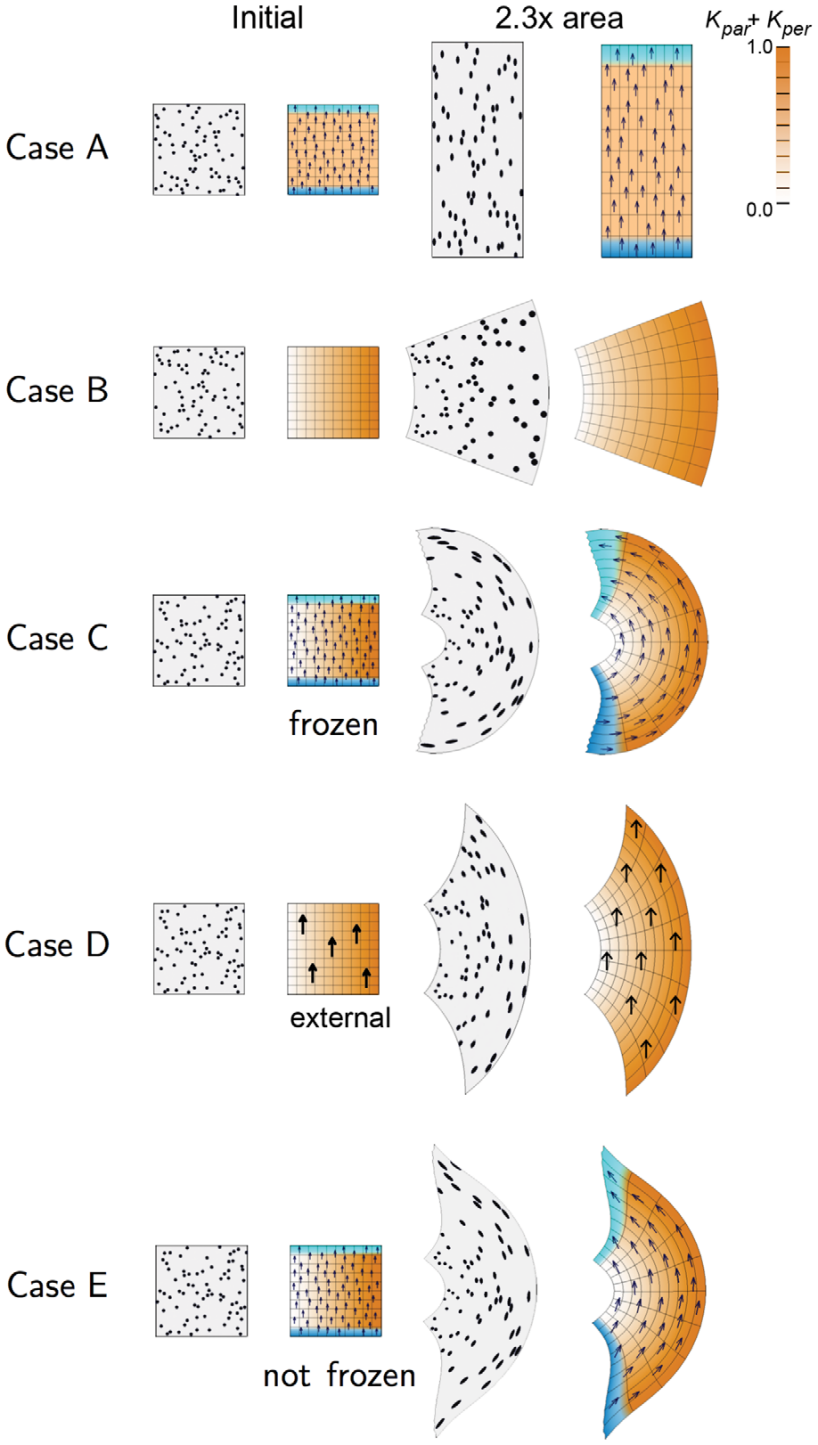
2D growth patterns with uniform POL gradient. Column 1 shows the initial state drawn with randomly scattered circular marked clones. Column 2 shows the initial state drawn with a regular grid and coloured to show areal specified growth rate (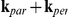, orange), POL gradient direction (arrows), 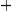
organiser (dark blue), and 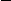
organiser (cyan). Columns 3 and 4 show the state after growth for a certain period. In Cases A, C, the POL gradient, once formed is no longer modified through propagation and deforms with the canvas. In Cases D, the POL gradient is held vertically by an external system. In Case E the POL continues to diffuse so the gradient is continually updated as the shape changes during growth. Deformations of the grid can be compared with the transformations of shape described in [33]. (Mesh of 3200 elements, growth magnitudes around 1 per unit time, 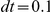, runtime 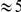 min for each example. 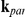 has arbitrary units.).

A key feature of the polarity-based axiality system is that orientation and growth rates can be specified independently and then combined in various ways. This combinatorial aspect is unlike the stress-based axiality system where orientations can only be specified once stresses have been generated in the tissue. These stresses will depend on the pattern of specified growth rates and the geometry of the tissue. To illustrate the combinatorial interactions within a polarity-based axiality system we first model simple anisotropic growth (Case A) and differential isotropic growth (Case B) separately. We then combine them in different ways (Cases C-I). We use an initially square canvas marked with black discs (simulating cells that produce marked clones) and a grid to show the geometrical transformations [33]. In all Cases the total areal increase (accumulated growth) is the same. The state of the canvas before and after growth is illustrated in Figure 1 for each Case.

Case A: Uniform polarity field with spatially uniform anisotropic specified growth rates. A gradient of POL is established during the setup phase through two organisers, 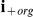 ( 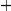
organiser ) and 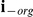 (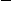
organiser ) at the bottom and top boundaries respectively. The PRN involves 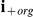 promoting production of POL while 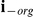 promotes degradation of POL, forming a proximodistal gradient of POL (arrows). After the setup phase the POL gradient is frozen (fixed to the canvas so that the gradient deforms with the canvas). An identity factor 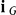 is expressed uniformly throughout the canvas. The KRN is 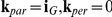 (the value of 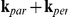 is indicated by the intensity of orange). The resultant growth transforms the square into a vertically stretched rectangle. The black discs become vertically oriented ellipses. The specified growth pattern underlying this transformation is straightforward to implement using the polarity-based axiality system. By contrast, an stress-based axiality system would require an additional step that generates vertically oriented stresses and thus an additional deformation. Moreover, the pattern of stresses would need to be maintained during growth unless there was a mechanism for fixing the axiality.

Case B: Spatially varying isotropic specified growth rates. Differential growth is achieved by promotion of specified growth rates towards the right side of the square. This involves establishing an identity factor 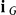 during the setup phase that is most strongly expressed along the right edge from where it declines gradually. The KRN involves 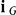 promoting the specified growth rates equally in all directions (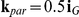, 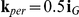). This leads to a gradient of locally isotropic specified growth that increases from left to right. The overall result is a curved fan. Curvature is not directly specified but arises through differential growth and mechanical constraints inherent in the canvas.

Case C: A combination of Cases A and B: uniform polarity field with spatially varying anisotropic specified growth rates. The PRN and KRN are the same as in Case A while the pattern of 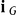 is the same as in Case B. That is, specified growth rate is oriented parallel to the POL gradient and increases towards the right. The result is a convex fan with much stronger curvature than Case B. Thus anisotropic specified growth, which on its own produces no curvature (Case A), reinforces the curvature arising through differential growth. In principle this reinforcement may arise from two causes. 1) Because there is no 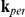, the gradient in 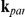 is greater than in Case B. 2) Because polarity is local, the directions of specified growth rotates with the canvas, enhancing curvature. To separate the contributions of these two components, we fix the direction of specified growth by using an external (global) frame of reference, as shown in Case D.

Case D: A combination of Cases A and B but using an external field to specify growth orientations. The gradient of POL is determined by an external frame of reference (y axis) instead of being embedded in the tissue. Biologically, external polarity information could be obtained from, for example, the effect of gravity. The result is a fan with reduced curvature compared to Case C. Note that ellipse orientations still deviate from the vertical because, even though growth is specified to be vertical, at each step mechanical constraints force the canvas to curve. The enhanced curvature of Case C over Case D reveals the contribution of orientations being specified internally (2) rather than externally (2). Another way of reducing curvature is by using a local polarity field that re-adjusts dynamically as the structure grows, as will be shown in Case E.

Case E: The same model as Case C but allowing POL to continue diffusing rather than being frozen after the setup phase. As with Case D, the resulting curvature is less than Case C, particularly near the extremities. This is because growth orientations turn less near the extreme positions of the canvas.

**Figure 2:**
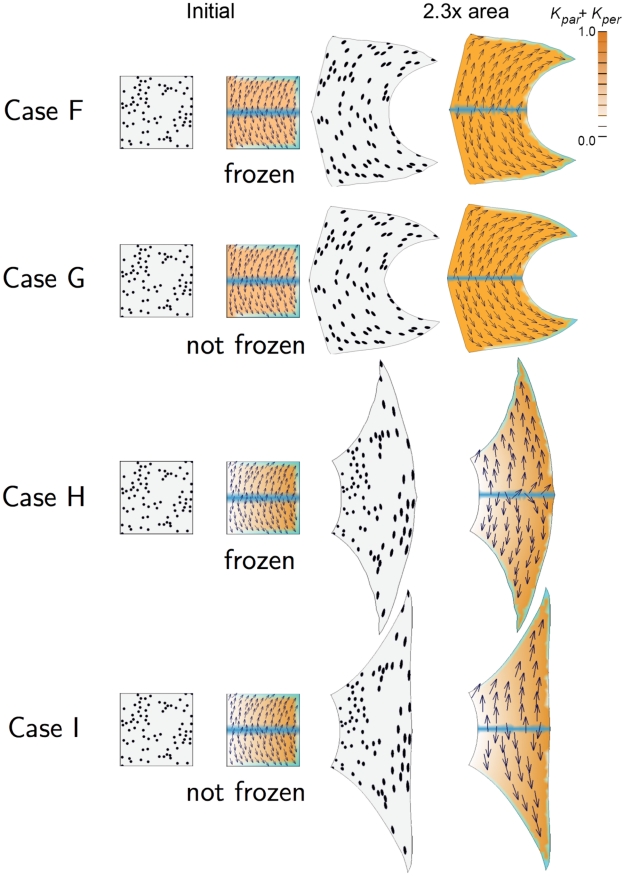
2D growth patterns with non-uniform POL gradient. Colours and symbols as for Figure 1.

The previous Cases considered uniform polarity fields with differential growth. This raises the question of how non-uniform polarity fields may influence shape. We first consider these when combined with uniform growth rates (Cases F and G, Figure 2) and then with differential growth rates (Cases H and I, Figure 2).

Case F: Similar to Case A but setting a spatially varying polarity field. A gradient of POL is established during the setup phase by 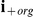, which is expressed along the horizontal midline, and 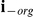, which is expressed in the top, bottom, and right edges, increasing toward the right corners. The resulting POL gradient is shown by the arrows. The polarity field is frozen (fixed to the canvas) after the setup phase. The distribution of 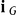 is spatially uniform as in Case A. Growth at the top and bottom edges is oriented by the 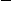
organisers producing a strongly concave right edge. Thus, curvature is generated as a result of non-uniform specified orientations of growth. The curvature is even stronger if the polarity field is not frozen after the setup period, as shown in Case G.

Case G: Similar to Case F but allowing POL to continue diffusing rather than being frozen after the setup phase. The result is more concave than Case F. This is because of feedback between canvas geometry and the polarity field. We now look at the effect of introducing differential growth rates.

Case H: Similar to Case F but with a gradient of specified growth rate. The PRN is the same as Case F leading to a polarity field pointing to the right corners. The KRN and distribution of 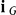 are the same as Case C leading to increasing values of 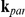 towards the right edge. The result is intermediate between Case C and Case F because the diagonally specified growth orientations counteract the curvature induced by differential growth. Thus, unlike Case C where local specification of orientation reinforces tissue curvature, here it antagonises curvature. This effect is still stronger when the POL gradient is not frozen as shown in Case I.

Case I: Similar to Case H but allowing POL to continue diffusing rather than being frozen after the setup phase. The right edge grows to be almost vertical showing that an appropriate specified local polarity can antagonise curvature arising from differential growth (Case B).

The main conclusion to emerge from Cases A to I is that the ability to combine specified growth rates with separately specified orientations provides an effective control mechanism for generating shape transformations. The shapes that emerge reflect interactions between specified orientations, differential growth and mechanical constraints. Depending on the spatial distribution of organisers and the dynamics of polarity propagation, tissue polarity can reinforce or antagonise curvatures resulting from differential growth or may generate curvature even in the context of uniform growth. So far we have only considered combinatorial interactions within the plane of the canvas. We next consider deformations out of the plane.

### Interaction between growth and polarity (3D)

**Figure 3:**
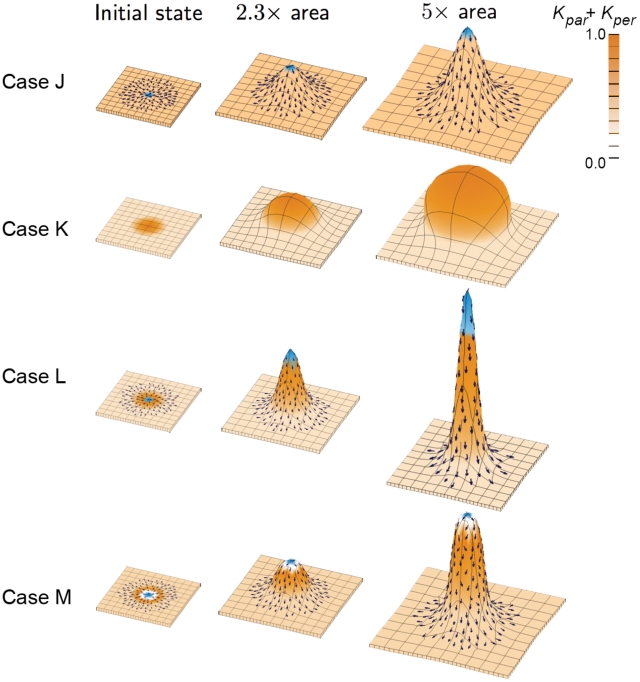
Shapes growing in 3D from a square canvas. In all cases there is a background specified growth rate (light orange) and each column shows the result of growing to a given multiple of the initial area. Symbols and colour coding as for Figure 1. (Mesh of 1800–2600 elements, growth magnitudes around 1 per unit time, 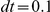, runtime 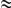 5 to 8 min for each example. 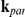 has arbitrary units.).

**Figure 4:**
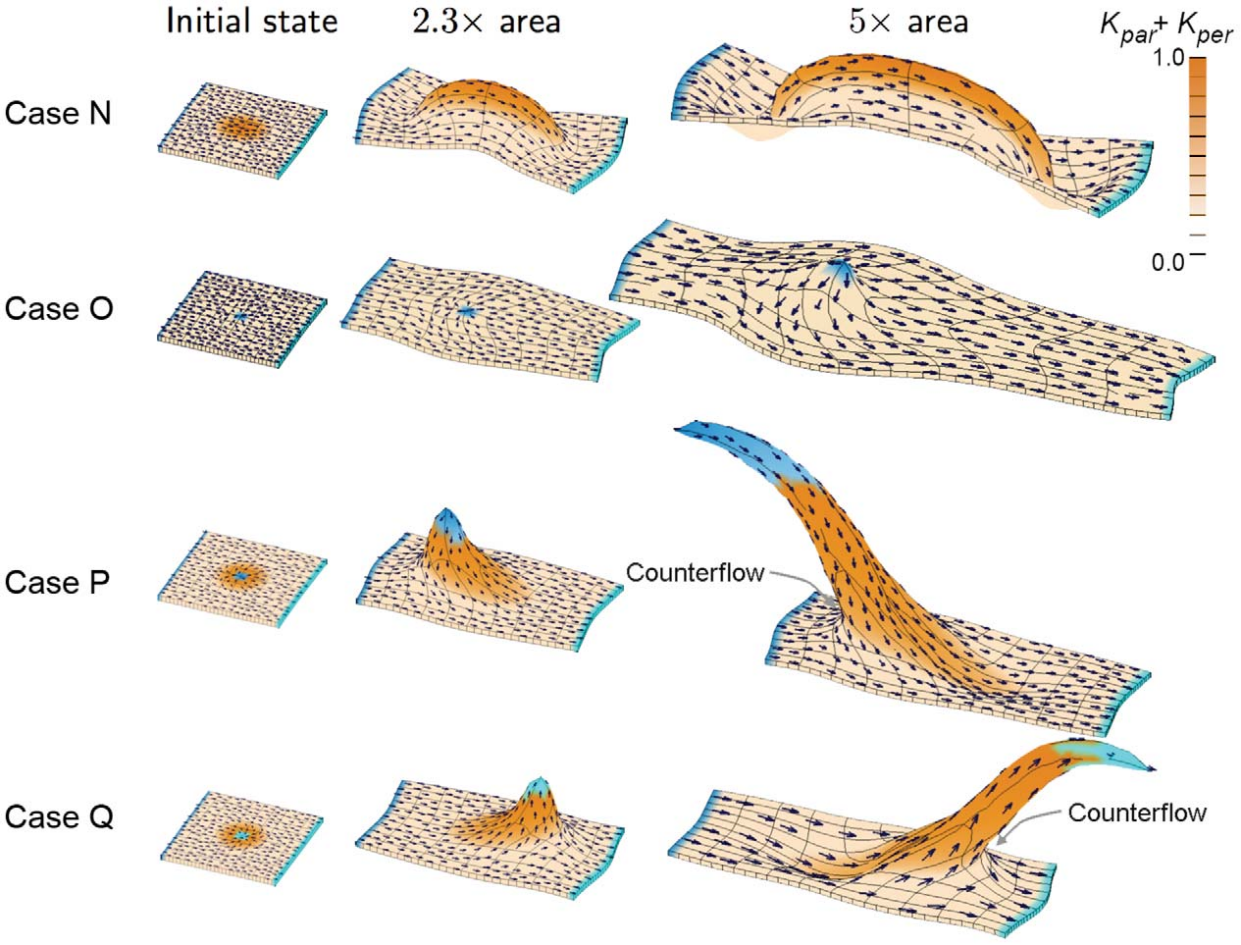
Shapes growing in 3D with superimposed gradients of POL. Symbols, colour coding, and execution parameters as for Figure 3.

We again consider a series of simplified cases (Figures 3 and 4) in which polarity and differential growth are treated separately (Cases J, K, O) and in combination (Cases L, M, N, P, Q). In each Case the up-down symmetry is broken by the centre of the initial canvas being slightly bowed upward. To simulate the presence of tissue beyond the boundaries of the initial canvas, the edges of tissue are prevented from moving or rotating out of the plane.

Case J: Spatially varying specified orientation with a uniform areal growth rate. The PRN involves an organiser (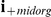), expressed in the middle of the canvas (blue). An outer region is defined by 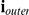 which keeps POL levels at zero. This leads to a divergent polarity field near the centre (arrows). POL continues to diffuse after the setup phase (i.e. the gradient is not frozen). 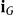 is spatially uniform as in Case A. The KRN involves anisotropic growth in the polarised region (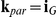 and 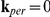). By default, growth is isotropic where the POL gradient is zero (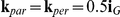). The result is a small spike. As with Case F tissue curvature has arisen through variations in specified growth direction even when areal growth rate is uniform. However, in Case J the curvature occurs out of the plane as well as in the plane.

Case K: Spatially varying isotropic specified growth rates. During the setup phase 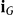 is established in the centre of the canvas from where it declines in a graded fashion. As with Case B, the KRN has 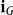 setting the specified growth rates, 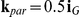, 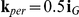. This leads to a gradient of locally isotropic specified growth rate that increases towards the centre. The result is a puffball-like central bulge exhibiting curvature both in and out of the plane of the canvas. The rounder shape compared to Case K illustrates the limitations of isotropic specified growth in creating elongated outgrowths. However, by combinging Cases J and K the outgrowth can be further exaggerated as shown in Case L.

Case L: Spatially varying anisotropic specified growth rates (combining Case J and K). The PRN and KRN are the same as Case J leading to radially directed growth. The distribution of 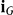 is the same as Case K leading to increased anisotropic specified growth towards the centre. The result is a tall central spike with a sharp tip showing how differential growth and anisotropy act in combination. In many biological structures, such as a growing plant apex, protrusions have rounded tips rather than sharp points. This can be achieved by reducing growth in the central region, as shown in Case M.

Case M: Spatially varying anisotropic specified growth rates with a central region of no growth. This is similar to Case L, except that additional identity factor sets 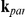 and 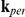 to zero in a small central region. The final shape is a rounded projection similar to what might be observed in a plant apex. Such a model is also consistent with the observation that growth rates tend to be lower in the central region of plant apices [12].

We conclude that a range of outgrowths can be readily obtained by combining specified growth rates and orientations. As for the 2D cases, deformations lead to changes in orientations of the polarity field which feed back to influence further deformations. So far we have considered the effects of uniform and divergent polarity fields. A further elaboration is to combine these two as illustrated in Figure 4.

### Interaction of polarity fields (3D)

Case N: A uniform polarity field with spatially varying anisotropic specified growth rates (combination of Cases A and K). The PRN and KRN are the same as in Case A leading to a left-right polarity field and anisotropic growth. The pattern of 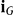 is the same as in Case K leading to enhanced growth in the centre. POL continues to diffuse after setup. The result is a thin bulge with grooves at each end. As with Case C, the polarity field is acting as a modulator rather than generator of curvature (no curvature is produced by the polarity field when combined with uniform anisotropic growth, Case A). We next look at the effect of combining the polarity fields in Cases A and J.

Case O: Interacting polarity fields with spatially uniform anisotropic specified growth rates (combination of Cases A and J). The PRN and KRN are the same as Case A except that the additional organiser from Case J is included. The new 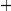
organiser distorts the polarity field shown in Case N inducing a saddle point upstream. As result following growth, the canvas widens slightly in the centre and forms a central ripple. Thus, as with Cases F and J, some curvature arises even with uniform specified areal growth rates. Next we combine this polarity field with centrally increased specified growth rates.

Case P: A combined polarity field with spatially varying anisotropic specified growth rates (combination of Cases K and O). The PRN and KRN are the same as Case O while the pattern of 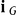 is the same as Case K. The result is an asymmetric spur reflecting the interactions between tissue polarity and growth. The asymmetry arises because the POL gradient generated by the central organiser flows in the same direction as the background POL gradient on one side but in the opposite direction on the other, creating a region of counterflow (arrowed). Disorganisation of growth in the counterflow region reduces growth along the main axis of the tissue. Asymmetry induced in this way is a feature of simple polarity-based axiality systems that would not occur in simple stress-based axiality systems. The orientation of the spur can be reversed by using a 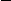
organiser instead of a 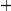
organiser in the centre as shown in Case Q.

Case Q: A combined polarity field with spatially varying anisotropic specified growth rates. The PRN and KRN and the pattern of 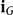 are the same as Case P except that the central 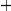
organiser is replaced by a 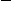
organiser. This time the asymmetric spur points in the opposite direction to Case P because the counterflow region is on the other side.

We conclude that combining polarity fields provides a further richness by generating asymmetries. The above Cases illustrate some basic combinatorial interactions between polarity and growth. To see how the same principles may apply to a biological example, we consider a simplified model of the Snapdragon corolla tube.

### Simplified model of the Snapdragon tube

To simplify the Snapdragon tube we assume the initial canvas comprises an initial cylindrical canvas closed at one end. As a first step we study locally isotropic specified growth (Case R) and then explore the effect of introducing specified anisotropic growth (Cases S and T).

Case R: Spatially varying isotropic specified growth rates. An early step in the development of the Snapdragon flower is arching over of the tube through differential growth. We simplify this process by restricting growth rates in opposite regions of the cylinder and also at the base. This is achieved by having a general background level of 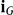 which is inhibited in the base by 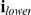 and is also inhibited by a diffusing signal 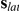 which is generated along opposite sides of the cylinder by 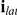. In this Case, specified growth is isotropic, 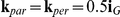.

**Figure 5:**
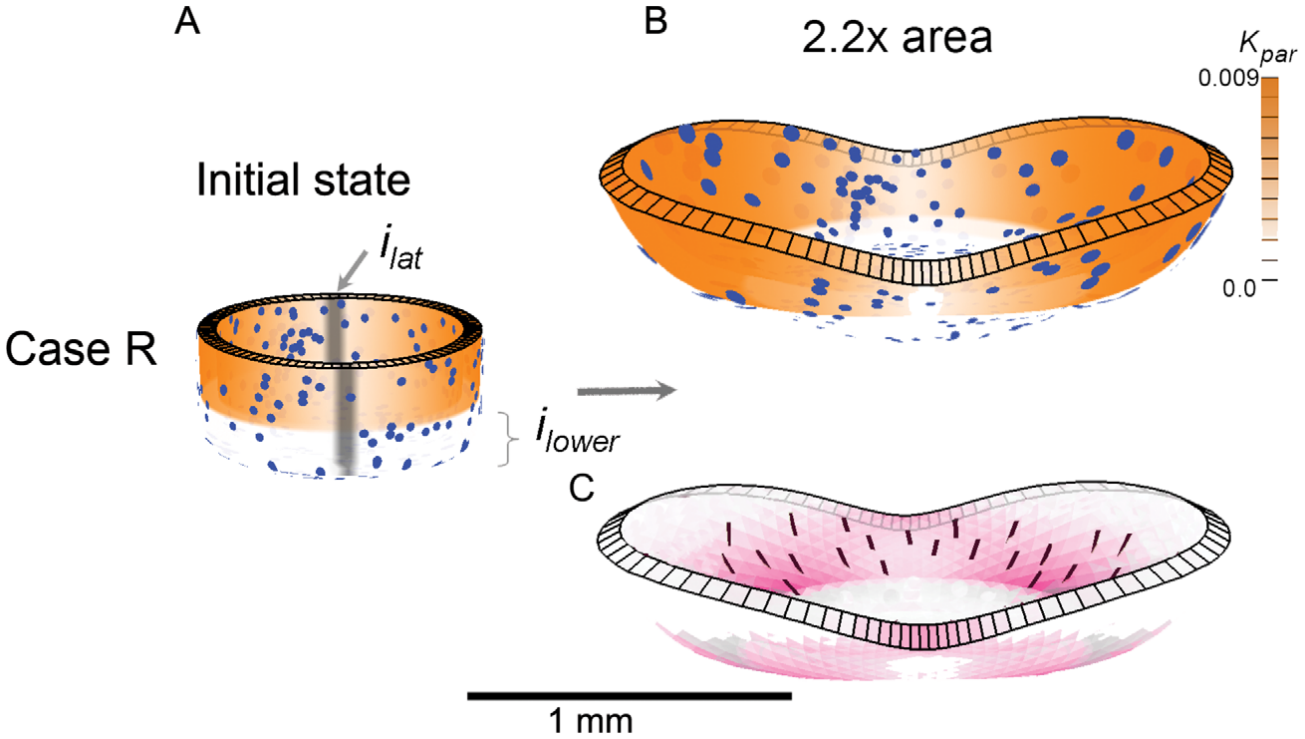
Case R: Spatially varying isotropic specified growth rates. (A) Initial shape with three regions, 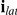 and 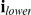. Orange colour denotes the value of specified areal growth. The initially circular discs monitor local shape changes. (B) Shape after growing to 2.2x the area. (C) As (B) but showing regions of resultant anisotropic growth (magenta) and its orientation (lines). (Mesh of 5600 elements, growth magnitudes around 0.018 per unit time, 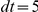, runtime 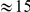 min for each example. 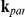 has arbitrary units. Vertices of the base are fixed in the Z-axis.) (A movie of this development is in ‘Video S1’.).

The result is a ballooned out bowl (Figure 5B,C) rather than an arched over tube. Some areas of the canvas near the base show anisotropic resultant growth, evident from elongated ellipses. This is shown more clearly Figure 5 C where the principal directions of resultant growth (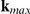) are shown with short lines and the rate of anisotropic growth (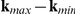) is shown in magenta. As with curvature, resultant anisotropy is not specified directly but arises through the interaction between differential growth and mechanical constraints. However, the pattern and extent of resultant anisotropy is inconsistent with experimental observations of clones in the Snapdragon tube, which are highly elongated along the proximodistal axis [31]. To address this discrepency we introduce specified anisotropic growth through a polarity field as shown in Case S.

**Figure 6:**
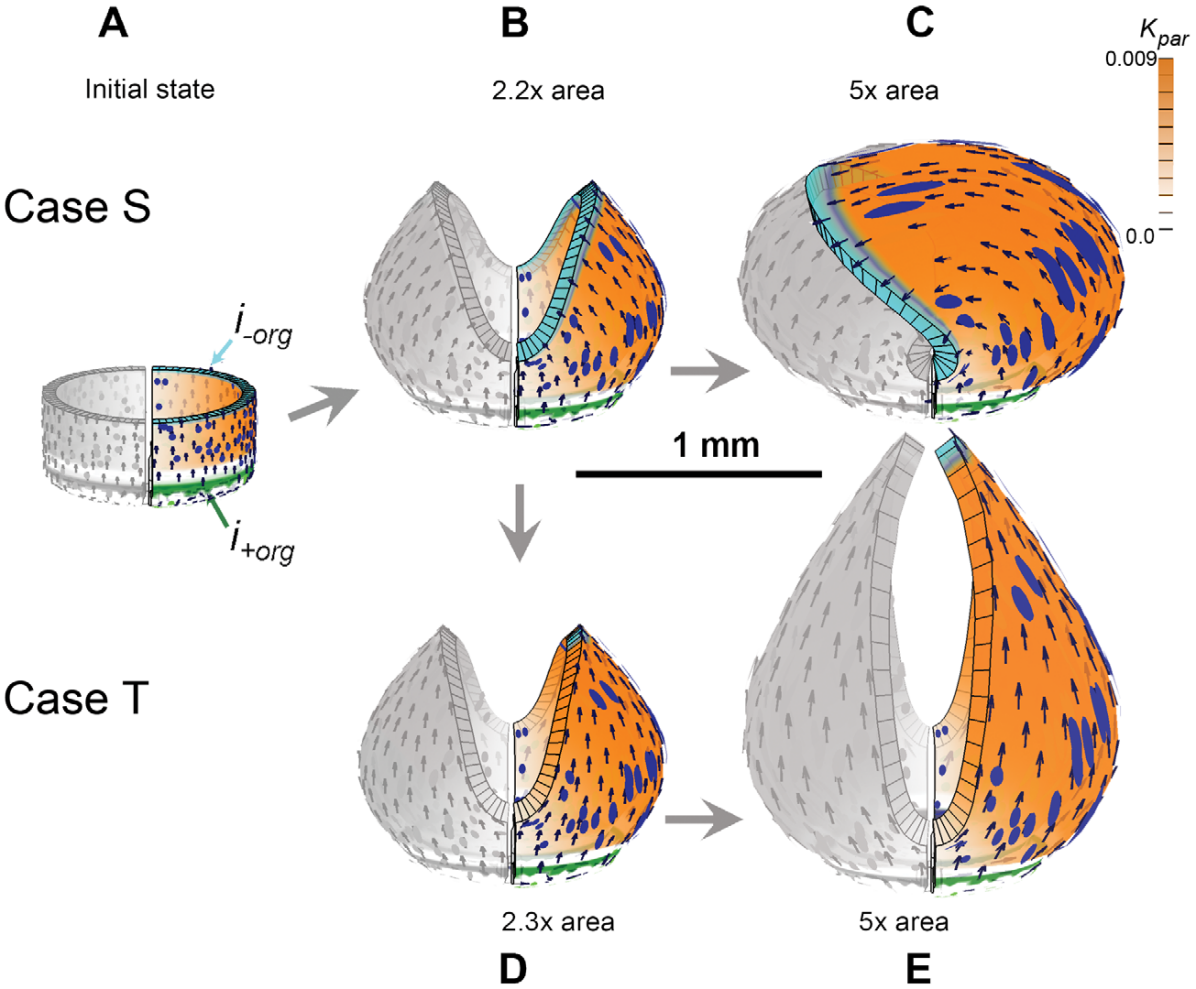
Cases S and T: An initially uniform polarity field with spatially varying anisotropic specified growth rates. (A) Initial shape with arrows showing proximodistal gradient of POL organised by the green and cyan regions (bottom and top) jonly half of the tube is shown colour-coded. Orange colour denotes the value of 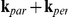. (B) Case S. At 2.2x areal growth the sides are arching over. Blue ellipses (induced as circles in initial state) show regions of local anisotropic growth. (C) Arching continues and at 5x areal growth the two sides overlap (there is no collision detection in our current software). (D) Case T. At 2.2x areal growth the distal organiser (cyan) is spatially redistributed to create two small patches causing the orientation of growth to change (arrows) and growth continues upwards (E). (Mesh of 5600 elements, growth magnitudes around 0.018 per unit time, 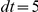, runtime 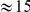 min for each example. 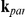 has arbitrary units. Vertices of the base are fixed in the Z-axis.) (Movies of these developments, C and D, are in ‘Video S2’ and ‘Video S3’.).

Case S: A uniform polarity field with spatially varying anisotropic specified growth rates. A gradient of POL is established through two organisers, 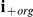 and 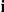-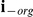 at the base and rim respectively (Figure 5A, arrows). The KRN is the same as Case R except that the specified growth rate is now anisotropic, 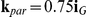 and 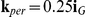. Compared to the output from Case R, the sides of the cylinder curve towards each other rather than ballooning outwards (Figure 6 B). Thus, introducing specified anisotropy has a major effect, leading to a more closed shape. It also generates much more elongated clones matching experimental observations. However, continuation of growth leads to the two sides arching further (Figure 6 C) rather than creating the elongated shape that is observed experimentally. (In our implementation which does not currently include collision detection the two sides grow through each other. For clarity we therefore only show one side in Figure 6.) To address this discrepancy, we exploit the potential to reorient growth within the GPT-framework by modulating the polarity field as shown in Case T.

Case T: Initially the same as Case S followed by reorientation of tissue polarity. There are two phases of growth, early and late. During the early phase the cylinder grows as in Case S. At the start of the late phase, the polarity field is modulated by restricting the spatial region of the 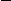
organiser. This is achieved by activating an identity factor 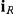 in the lateral regions of the cylinder which inhibits 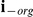, restricting the distal organisers (cyan) to small regions at the apex of each arch (Figure 6D,E). The reoriention of polarity leads to vertical elongation of the arch rims, maintaining the closed shape, rather than the sides continuing to arch over. This captures an essential feature of Snapdragon corolla tube growth.

## Discussion

We model growth through the accumulation of a series of small deformations of the tissue (canvas). Stresses are generated during the process as the canvas is mechanically interconnected. This may lead to anisotropic resultant growth even when growth is specified to be isotropic (e.g. Case R). In principle, such resultant stresses could be used, through stress-based axiality, to orient all forms of anisotropic growth. However, this would mean that specified orientations of growth would be dependent on differential rates of growth, precluding the possibility of independent control. By contrast, we show how a polarity-based axiality system allows diverse forms to be generated through combinatorial interactions between specified orientations and rates of growth.

In this system, a key element in controlling growth orientations is the distribution of polarity organisers. These are of two types, 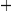 or 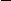, allowing polarity fields to be anchored at both ends. Even when specified anisotropic growth is uniform over the canvas, a range of forms can be generated by varying the pattern of organisers. For example, starting from an initial square canvas it is possible to generate rectangles (Case A), concavities (Case F), small spikes (Case J) and ripples (Case O). In these Cases polarity was fixed after a setup period. Biologically, this would correspond to an initial period when polarity propagates across the tissue (when the tissue is small), followed by polarised cells maintaining their polarity and passing it on to their daughters. Another possibility is that polarity continues to propagate during growth leading to slight modifications of the resulting shape (compare Cases F and G).

The range of shapes may be greatly extended by combining polarity fields with differential growth rates. For example, tissue polarity may reinforce or antagonise curvature arising through differential growth (Cases C and I). Both aspects are incorporated into the growing Snapdragon tube - reinforcement of curvature during the early phase leading to arching over (Case S), followed by antagonism of curvature leading to straightening (Cases T). It is also straightforward to generate extended outgrowths and apices by combining a single organiser with enhanced growth (Cases L and M). A further feature of polarity-based axiality systems is the emergence of asymmetries through interactions between polarity fields. For example, asymmetric spurs may arise because of counterflowing polarity on one side (Cases P and Q). The asymmetry of the outgrowths in these Cases results from the underlying polarity interactions and would not have arisen from a simple system with only stress-based axiality.

**Figure 7:**
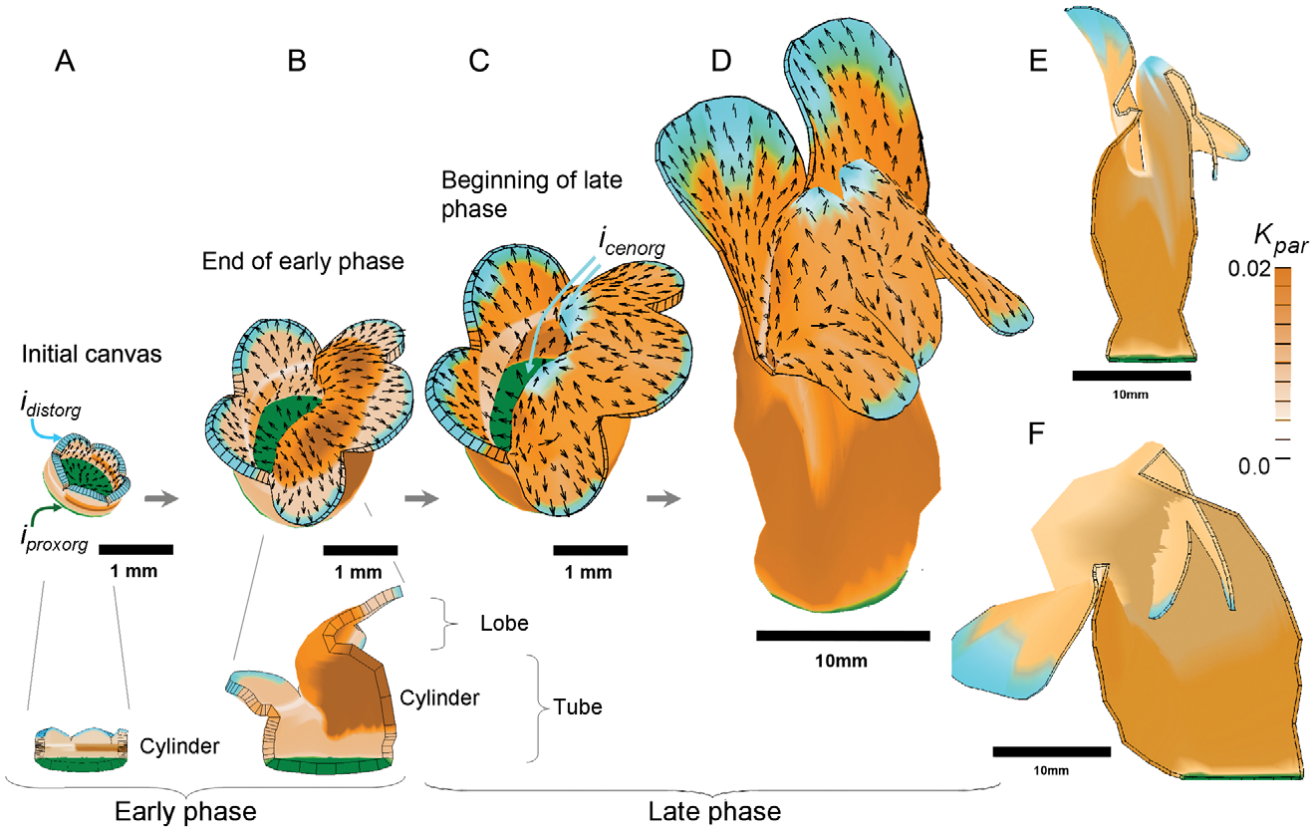
Patterns of growth in the Snapdragon model [**31**]. (A) Initial canvas showing organisers of polarity, 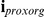 and 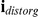 (green and cyan respectively) and cylindrical shape. Orange indicates growth rate parallel to the POL gradient. (B) By the end of the early growth phase, extra ventral growth (dark orange) creates an arch (as in Figure 6). (C) At the beginning of the late phase 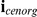 is formed and anisotropic growth has reoriented along the new axis (arrows show polariser gradient that now points towards 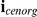, cyan). (D) Adult shape in which the ventral arch has grown upwards (see Section in Fig.1C). (E) Vertical section through adult shape. (F) Similar view of the same model except that anisotropic growth is not reoriented. (Mesh of 3000 elements, growth magnitudes around 0.003 per unit time, 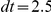 hours, runtime 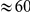 min for each example. 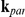 has arbitrary units.) (Movies of these developments, B, C, E, F, are in ‘Video S4’, ‘Video S5’, ‘Video S6’, ‘Video S7’.).

In these examples only a few organisers are needed to achieve major shape transformations. To test whether the same simplicity might underly more complex biological transformations, we modelled growth of the Snapdragon flower [31]. This model is constrained by a range of experimental data. The expression pattern of the genes DIV, CYC, DICH and RAD are set according to experimental observations. The model has to not only account for the wild-type phenotype but also double (*cyc, dich)* and triple (cyc, dich, div) mutants. The model is also constrained by the observed changes in 3D shape determined by optical projection tomography at several developmental stages. In addition the pattern of growth rates and directions over each model petal need to be similar to those observed by clonal analysis. The model starts with an initial cylindrical canvas with five lobes and a proximodistal pattern of polarities established through two polarity organisers (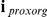 and 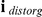) (Figure 7 A). During the early phase of growth the ventral region of the tube arches over through differential anisotropic growth. To account for the observed pattern of clones a third organiser (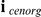) is introduced (Figure 7 C). In the absence of this organiser the tube bulges out (Figure 7 F) similar to what happens in the simplified corolla with no reorientation of growth (Case S). However, with the introduction of the organiser the tube automatically straightens out during later stages, consistent with experimental observations. Thus, this biologically relevant case provides evidence for three organisers underlying major shape transformations and growth dynamics. In the Snapdragon model, the reorientation of growth is under the control of DIV, a gene that encodes a Myb-like transcription factor that affects flower shape and symmetry [31]. As well as its effect on organiser activity, DIV also influences growth rates. Thus, although rates and orientations of growth are specified separately in the model they can be regulated by a common gene.

The polarity-based axiality system has the flexibility to account for global shape changes, observed growth patterns and clones without invoking large numbers of polarity organisers. This alone does not demonstrate the validity of invoking tissue polarity for the control of growth orientations. Nevertheless, tissue polarity is commonly observed in animals, for example, polarised cell movements [27] and in plants where the polar distribution of molecules within cells, such as PIN auxin transporters, suggests that cell polarity is also common [23], [24]. It has also been proposed that an auxin concentration maximum at a vascular boundary in the root tip establishes a distal polarity organiser in the root [20]. The GPT-framework allows hypotheses on polarity-based axiality growth to be established that can be subjected to further tests such as mechanical or genetic perturbations. The Snapdragon model, for example, was evaluated against predictions of shapes of multiple mutants not used to build the model [31], [32]. The results showed a good, quantitative, fit between predicted and observed shapes. The model also makes important predictions about the location of polarity organisers. Polarity markers are predicted to show reversals (i.e. arrows pointing away or towards each other) at these locations.

In all our Cases we make the simplifying assumption that the tissue is linear over small deformations and has isotropic material properties. An elaboration of the GPT-framework would be to incorporate non-uniform properties, although this would also require these properties to be measured across the tissue during growth. The GPT-framework is consistent with current hypotheses regarding the mechanisms in which plant tissue grows under turgor pressure through the loosening and formation of bonds (Theorems 1 and 2, Methods). Loosening bonds in the cell wall allows the tissue to grow. If new material is inserted that restores the properties of the cell wall then the residual strain returns to zero (‘snip and fill’ [31]). Biologically this would require some form of feedback between resultant stresses (or strains) and cellular properties [34]. Feedback from stresses to microtubule patterns has been proposed [19], and this can be interpreted as reflecting the need to dissipate residual stresses rather than being the primary way of orienting specified growth. Cutting provides a convenient experimental way to evaluate the extent to which residual stresses accumulate or dissipate in a given biological system. Often they accumulate in certain regions in later developmental stages. For example, the dorsal and ventral petals of the adult Snapdragon flower press against each other holding the flower shut (not a part of the model in Green *et al* [*31*] *).* The observation that the accumulation of residuals varies systematically from region to region suggests that the process of dissipating or accumulating residuals is under genetic control. Stresses that are accumulated can be modelled with the GPT-framework and, to enable direct comparison with experimental results, the resulting shapes can be cut allowing the structure to spring into a new shape.

**Figure 8:**
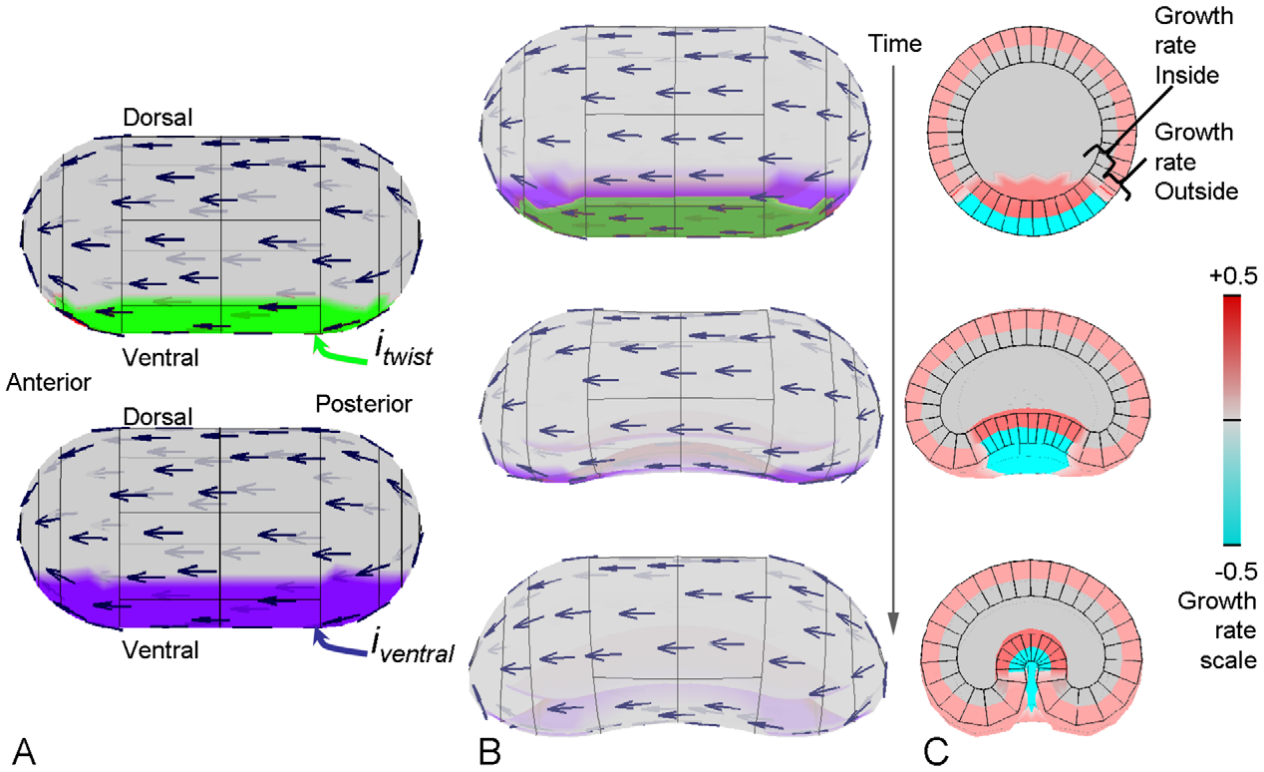
Invagination in the developing *Drosophila* embryo. (A) Initial pattern of 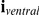 and 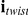 on a shape that is polarised from posterior to anterior (arrows). (B) Side view of the developing embryo. The patterns become occluded as the furrow develops. (C) Transverse section of embryo showing colours representing relative specified growth rates perpendicular to the polariser gradient on the internal and external faces. The furrow is produced by a shrinkage on the outside coupled with an expansion on the inside and a net shrinkage in the ventral region (specified by 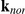). Cyan shows negative specified growth on the outside and dark red shows positive growth on the inside. The images are all to the same scale.

The GPT-framework assumes that regions (e.g. cells) in a tissue do not slide or move past each other. This is valid for plants [35], making them particularly appropriate for this approach. The GPT-framework may also be applicable to some aspects of animal development. For example, finite element models have been used to capture deformations during *Drosophila* ventral furrow formation driven by apical constriction and apicobasal elongation of cells [8]. Comparable deformations can also be generated using GPT-framework by using a posterior-anterior polarity field [36] and incorporating negative growth (contraction) on one side of the canvas (Figure 8). Although this model does not incorporate all biologically relevant features such as constraints of the external vitelline membrane, it illustrates the flexibility of the approach.

Clones generated in early wing development of *Drosophila* often stay as contiguous patches, indicating that connectivity is broadly maintained and extensive mixing of cells does not occur [37]. Greater cell mixing is observed for clones in developing mammalian tissues such as the heart or limb, although even in these cases cell movements are not sufficient to disrupt formation of clonal clusters or patches[38]. At the tissue scale it may therefore be reasonable to model many animal structures with the framework described here, particularly as orientated cell behaviours are thought to play a critical role [39], [40].

As well as multicellular tissues the canvas could represent a region of a plant or bacterial cell wall. By extension of the GPT-framework it may also be possible to capture the growth of compartments enclosed by a canvas (e.g. cells with their walls) or growth of a bulk solid. Thus, the GPT-framework provides a general approach that can be applied to growing tissues at many scales.

The GPT-framework with its assumption of tissue polarity as a key component of growth specification provides an economical way of generating diverse shapes and forms. We hypothesise that this combinatorial richness is not only computationally attractive but has also been exploited during evolution to generate a range of observed biological shapes.

## Materials and Methods

Various mathematical and computational methods [21] have been used to model tissue growth. These range in scale from detailed modelling of individual sections of cell wall to larger scale models treating the tissue as a continuous substance. The physical properties have been studied in terms of mass-spring models, elasticity theory of thin shells, and elasticity theory for solid volumes. Elasticity theory described here subsumes both classical linear elasticity theory and elastoplastic or viscoplastic theory for modelling solid flow.

In mass-spring models tissue is represented as a set of point masses linked by springs. De Boer [41] combines mass-spring modelling with the L-system formalism of [42] to describe a two-dimensional model of cellular growth. In these models, and in those of [13], [43], [44], the springs correspond to sections of cell wall, and the masses are where three or more springs meet. Growth is modelled by changing the resting length of the springs. The new equilibrium configuration is then computed by iteratively finding a state of minimum energy. There are empirically-based rules for deciding when cells should divide. These models are mainly limited to two-dimensional problems, although they have also been used to model model axisymmetric three-dimensional solid problems such as root tip growth. A problem with mass-spring modelling of continuous tissue (i.e. above the cellular scale) is that it is not trivial to design the model so that on a large scale, realistic elastic properties emerge. For example, a regular grid of springs is not geometrically isotropic.

For tissues which take the form of curved surfaces, thin in comparison with their extent, one can use thin shell theory (c.f. sheets of cells [45]). This is the branch of elasticity theory dealing with the mechanics of curved surfaces [46]–[48]. It is the limit of three-dimensional bulk elasticity theory as the thickness of the sheet tends to zero while retaining its bending stiffness properties. For surfaces which are extremely thin in comparison to their area, this has advantages for numerical computation over describing them by the methods of solid volume elasticity theory. The rippled edges of leaves have been modelled by this method as the mechanical consequence of faster growth at the edges [49], [50]. (Cf. Text S1, Case 14 and Video S8 [7], [51], [52].)

A third approach is to model biological structures as three-dimensional solid objects [19], [53]. This can be appropriate when tissue thickness is sufficiently large to make the thin shell approximation unnecessary. The method is analysed theoretically by Goriely and Ben Amar [54], who consider the general problem of describing the growth of elastic substances resulting from local growth fields and, by alternating a phase of growth without movement (that is, insertion of new material) over a small time interval and then allowing elastic relaxation, they show how growth over an extended period of time can be modelled. The net result is a visco-plastic deformation. It is this approach that is taken in the GPT-framework, and it has been extended to model both the extent and orientation of anisotropic growth.

### Calculating growth

The following theory covers the local specification of growth, how to compute the resulting growth given the mechanical properties of the canvas, how to handle residual growth, and how modelling using the GPT-framework relates to modelling growth in terms of turgor pressure and modifications to the mechanical properties of the cell walls.

**Figure 9:**
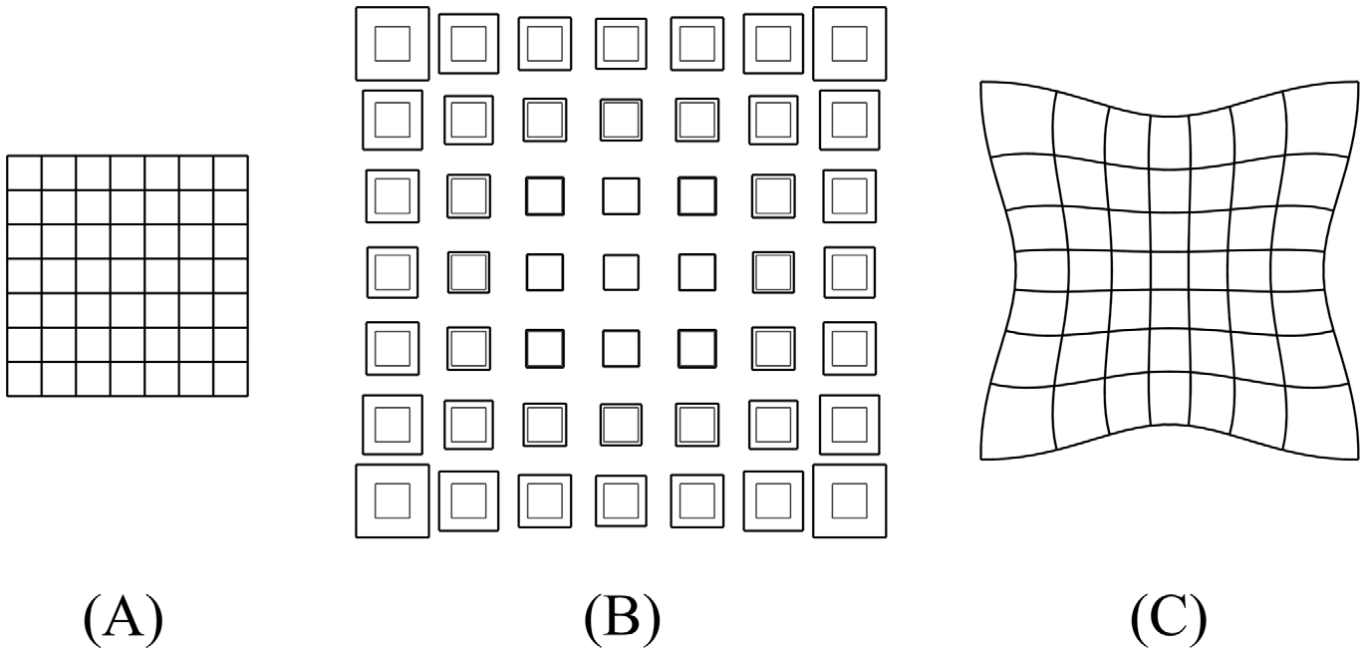
Specified and residual strain. (A) The initial state. (B) Exploded view of the specified growth of each tile, with the original sizes superimposed in grey. (C) The minimum-energy shape that results from the constraint of continuity.

We distinguish two types of growth, specified and resultant. Resultant growth is the growth that can be directly observed by tracking or clonal analysis. Specified growth is the growth that would happen to an element of the canvas if it grew in isolation. Resultant growth emerges as result of specified growth in different regions interacting through connected tissue. This is illustrated in Figure 9. Panel (A) shows the initial state of a square tissue, divided into a number of small tiles. If we apply a radially increasing field of locally isotropic growth, then in (B) we have an exploded view of how this would affect each tile individually, if it were not attached to its neighbours. It is clear that without some further deformation, these tiles cannot fit together into a continuous tissue without gaps. This conflict between the specified growth field and the continuity of the tissue leads to an equilibrium compromise between the two shown at (C). It is mathematically determined by the partial differential equations of elasticity theory, and numerically computed by the finite element method, both of which we shall briefly summarise.

### Describing resultant growth

Suppose that at a given time, each point at position 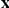 in a tissue is moving with an instantaneous velocity 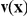. The resultant growth rate 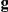 in the neighbourhood of 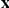 is the gradient of the velocity field 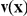 with respect to 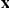. This is the second rank tensor field (a two-dimensional matrix at each point of the tissue) 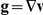 whose components are 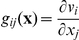, where 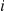 and 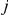 range from 1 to 3. This *velocity gradient* tensor represents both the change of shape and size and the rigid rotational motion of the material in the neighbourhood of the point 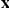. These are respectively its symmetric and skew-symmetric parts: 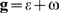, where 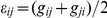 and 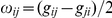. 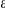 is called the *resultant strain rate* tensor field, and 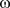 the *resultant vorticity*. The vorticity field describes the angular velocity at each point. When the vorticity component of a tensor field is zero, the field is called irrotational. To avoid subscripts we abbreviate the definition of 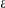 to 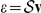, where 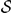 is the differential operator defined by 
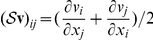
. The rate of resultant growth of the material in any particular direction 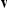 is the sum 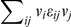.

Because the resultant strain rate tensor 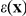 at a point 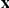 is symmetric, it can be diagonalised by suitably rotating the local frame of reference. The resulting three diagonal components are the principal rates of resultant growth, in three perpendicular directions. These are the eigenvalues of 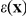, and the principal growth directions are parallel to its eigenvectors. The growth directions and rates will in general vary over the tissue.

### Calculating resultant growth from specified growth

To explain how resultant growth may be calculated from specified growth, it is convenient to think in terms of small displacements rather than velocities, by considering the effect over a small time 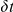. This is also how the computational implementation (to be discussed below) works, iterating through time in small steps 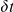. “Small” here means small enough that first-order approximations apply. In time 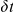 a velocity field 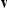 produces a small displacement field 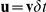, and a growth rate or strain rate tensor field produces an amount of growth or strain, which we shall denote by the same symbols as before.

At each point 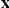 in the growing canvas, let 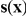 be a specified strain tensor at that point, being the product of a strain rate tensor by a small time 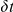. This is the growth that would occur in a small region around 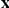 in time 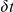 if it were mechanically isolated from the rest of the tissue. Let 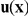 be the displacement field that will result from this pattern of growth if the tissue remains in mechanical equilibrium, and 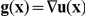 the associated growth tensor field. Except in some special cases, such as uniform isotropic growth, 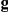 will differ from 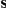. Even if the rotational component of 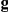 is ignored, its strain component 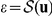 will still in general differ from 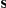: there may be no displacement field 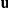 of which 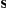 is the strain field. This is due to the constraint of physical continuity that we mentioned above. (For clarity, the amount of growth shown in Figure 9 has been made far greater than we would normally compute in a single time step.)

Physical continuity is expressed mathematically by the St. Venant compatibility constraints [55]. If 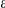 is a strain field of the form 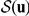, then it necessarily satisfies the following partial differential equation:
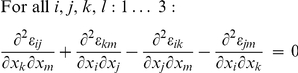
This can be verified by substituting 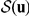 for 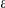 and (somewhat laboriously) finding that all of the terms in the resulting sum of third derivatives of components of 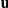 cancel out. It is a deeper result that the St. Venant conditions are sufficient for such a velocity field 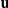 to exist.

If, on replacing 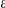 by 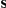 in the above equation, it fails to hold, then whatever deformation 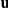 is applied, the material must remain in a state of frustration. There will be unrelieved residual strain given by 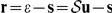. When the material is in mechanical equilibrium, the displacement field 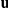 will be such as to minimise the energy contained in that residual strain. To calculate 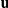, we use the principle of virtual work: if the material is in equilibrium, and any additional infinitesimal displacement 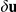 is applied, then it will do zero work against the stresses in the material ([56], ch. 2).

These stresses are given by a tensor field 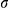 calculated from the strain and the elasticity properties by the constitutive equation of the material:
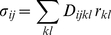
(1)The subscripts all range over the spatial dimensions 1–3. 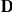 is the *elasticity tensor* or *stiffness tensor*, a 4th rank tensor field representing the elasticity properties of the substance [57]. The work done by any small strain 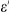 against any stress 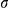 is 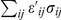, and the total work done for strain and stress fields is found by integrating this over the whole tissue.

This is the linear elastic constitutive model, which we are assuming to be valid for small strains. For some biological tissues this assumption may not be accurate, for example as noted in [58] for the mouse ventricle, which also notes that determining a more accurate constitutive model is experimentally challenging.

To avoid writing explicit summations, we shall adopt the notations that if 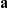 and 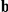 are second rank tensors and 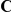 and 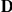 are fourth rank tensors, then:



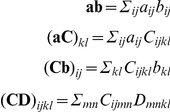





The work done by the strain 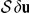 against the residual stress is then:
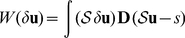
(2)where the integration is over the whole volume. For 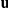 to be the equilibrium deformation we must have:
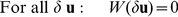
(3)


Except for degenerate situations (such as the initiation of buckling [59]), this determines 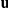 up to a rigid translation or rotation of the whole object.

We have omitted from equation 3 the possibility of external forces acting on the substance, since there are no such forces present in the applications used in this paper and the Snapdragon model [31]. Boundary conditions can also be applied which stipulate that some parts of the substance remain stationary. We describe how these are handled when we discuss numerical methods.

Both the specified growth field 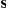 and the resultant strain field 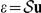 are by definition irrotational. However, the resultant growth field 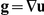 in general does include rotations. Leaving aside rigid rotations of the whole tissue, the relative rotations between different parts of the tissue are entirely determined by the irrotational tensor 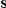. That is, relative rotations are caused solely by differential local growth and the continuity constraints, not by any explicit specification: rotations are always resultant, never specified.

Since 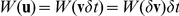, the whole analysis carries back to the description in terms of velocities, strain rates, and growth rates.

In plants, specified growth rates are always positive, but in animal tissue this is not always so. Both positive and negative growth rates in any direction can be handled computationally without difficulty. Figure 1 shows a simple model in which the shape changes with negligible change of volume.

**Figure 10:**
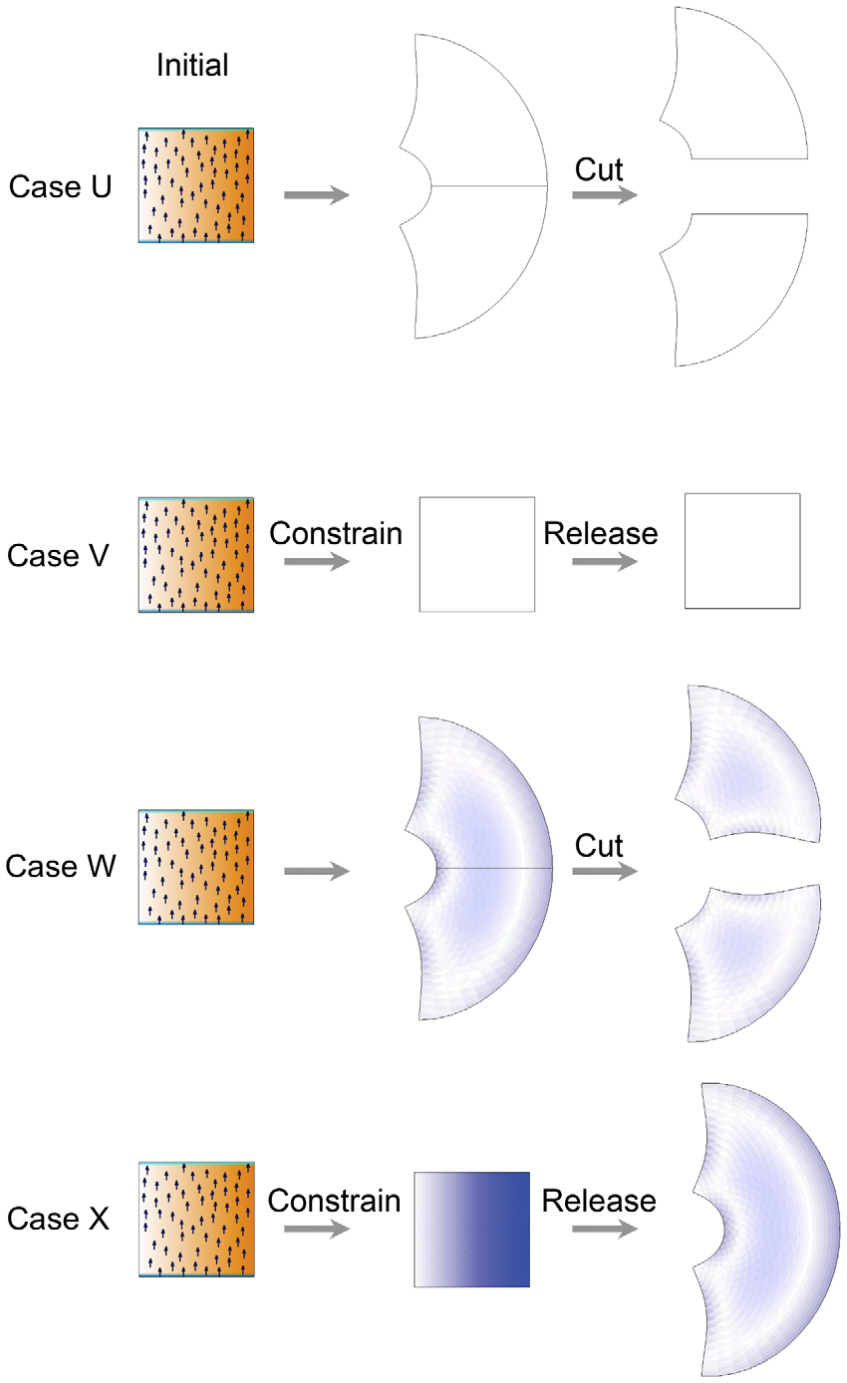
Comparing dissipating residual strains, Cases U and V with accumulating residual strains Cases W and X. The residual strain after growth is revealed in three ways: by colour (the residual strain is shown in blue); by cutting and re-equilibrating the canvas (Cases U and W); and by releasing constraints (Cases V and X). In Case U growth produces an arc but there is no accumulated strain - no colour - and there is no further change in shape on cutting. Likewise Case V. However, in Case W the shape changes on cutting and in Case X the shape change on releasing constraints. Both these changes reveal the accumulated strains.

The residual strain is given by the tensor 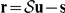, which is the symmetric part of the residual growth tensor 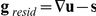. Most of the examples in this paper discard the residual strain after each time-step of the simulation. In biological terms this is consistent with the observations of [19] that imply a feedback mechanism that acts to absorb stresses. To illustrate the effect of discarding or retaining residual strains we consider several cases in which we cut the canvas after growth or constrain the canvas during growth and then release the constraint. We contrast the effect of discarding residual strains (Cases U and V) with accumulating strains (Cases W and X). These are illustrated in Figure 10.

Case U: Dissipation of residual strain with a non-uniform pattern of growth followed by cutting. This is identical to Case C in which residual strain is dissipated at each step. As expected, cutting the canvas induces no further changes of shape as there is no accumulated residual strain. We next show the result of constraining the canvas so that it cannot grow followed by releasing the constraint.

Case V: Dissipation of residual strain with a non-uniform pattern of growth that is constrained, followed by release. The model is the same as Case C except that all the boundary points are fixed during growth (column 2). When these constraints are released the shape does not change (column 3) as there is no accumulated residual strain. We next consider the effect of accumulating the residual strain.

Case W: Accumulating residual strain with non-uniform pattern of growth followed by cutting. The model is the same as Case C except that residual strain is accumulated at each step. The result is very similar to Case C but there is a small accumulated residual strain (column 2, blue). Cutting and allowing the canvas to relax releases some of this accumulated strain leading to a curve along the line of the cut compared to the straight line in Case U.

Case X: Accumulating residual strain with non-uniform pattern of growth that is constrained, followed by release. The model is the same as Case V except that strain is accumulated during growth (blue shows accumulated strain). Releasing the constraints allows a shape to emerge similar to Case W uncut.

**Figure 11:**
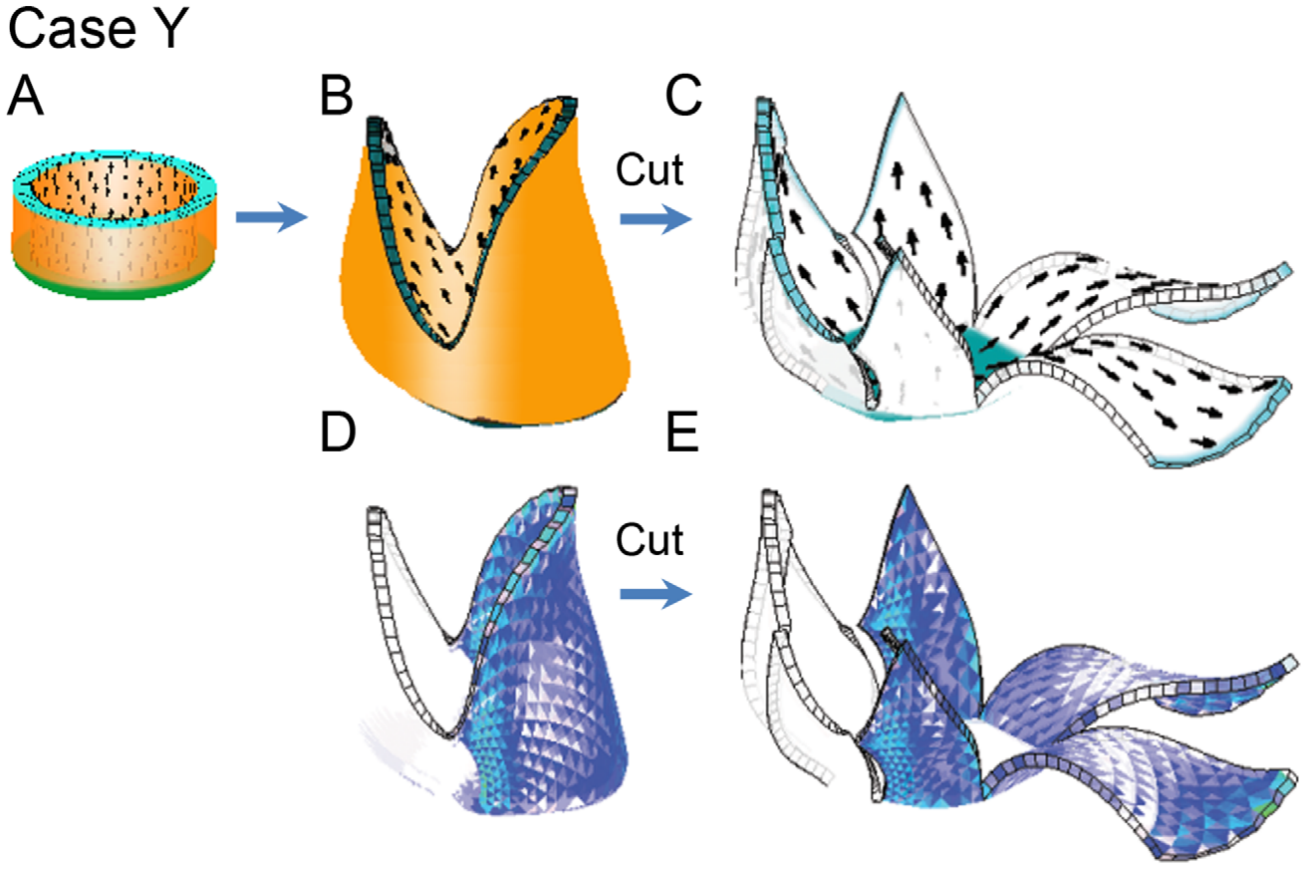
Relieving accumulated strain by cutting. (A, B) Shape grown similarly to Figure 6 E. The specified growth rate is shown in orange. (D) As for (B) but showing the accumulated residual-strain (blue). Strain is not retained on the left side and is fully retained on the right side (strain retention is controlled through the action of 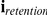 which is only active on the right side). (C, E) The result of turning off growth, making 8 vertical cuts in the mature shape and allowing the shape to re-stabilise.

We may also illustrate the effect of retaining residual strain with a 3D example. For this we use the simplified Snapdragon tube (Case T), but allow residual strain to accumulate on one side. This is illustrated in Figure 11.

Case Y: This is the same as Case T, except that residual strain is retained on the right side. Figure 11 B shows how the resultant shape of the right side differs from the left. The residual strain is shown in blue (Figure 11 D). A further difference between the two sides is revealed by making vertical cuts and allowing the mechanical system to relax to a new geometry (Figure 11 C,E). As expected, cutting makes little difference to the left side as there is little residual strain. However, the right side springs apart revealing some of the stored residual strain.

In the above examples the release of residual strain by cutting involves large displacements and rotations of the material. However, our computational methods are based on the linear elasticity theory of small displacements, and never directly solve large-displacement problems. The deformation resulting from the release of residual strain is, therefore, computed incrementally, by iteratively applying a small fraction of the residual strain, computing the resulting small deformation, transforming the remainder of the residual strain according to the new orientations of every part of the tissue, and repeating until an equilibrium is reached.

The stiffness tensor 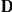 is a fourth-rank tensor, which in three-dimensional space has 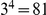 components at each point. However, it satisfies certain symmetry properties which imply that it has at most 21 independent components. For isotropic materials, further symmetries imply that 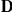 is determined by just two values: the bulk modulus 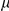 and Poisson’s ratio 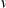. 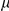 is the ratio of applied pressure to relative change in volume. We will see later (see TextS1, Equation (S1) *et seq.*) that its value is irrelevant for the calculations we require: it cancels out of the equations. When a block of material is compressed by external forces in one direction, Poisson’s ratio 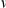 is the ratio of its transverse expansion to its longitudinal compression. In practice 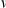 lies between 0 and 0.5. As the value approaches 0.5, while the bulk modulus is held constant, the resistance to unidirectional stretching and compression decreases towards zero. If the limit is instead approached by keeping the shear modulus constant, then the bulk modulus tends to infinity. In the former case, the material’s resistance to shears vanishes and it approaches the state of a liquid, while in the latter it approaches an incompressible solid with a finite elastic resistance to everywhere volume-preserving deformations.

However, since there are no applied forces (such as gravity) in our models, but only growth described as a change in the resting shape of the material, the difference is more apparent than real. The elasticity tensor 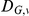 computed from 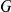 (the shear modulus) and 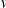 (Poisson’s ratio) is equal to the tensor 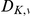 computed from 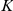 and 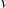 multiplied by 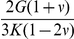. As mentioned above, any such factor in the elasticity tensor cancels out (Text S1, Equation (S1)), because all of the forces that we consider result from the material acting against itself. Both methods of computing the elasticity tensor for any value of 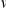 less than 0.5 give identical solutions to the equation, solutions which are independent of 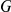 or 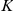. At exactly 0.5 the equations become highly degenerate, and a different analysis is required to calculate the physical behaviour in the limit. Any value above 0.5 is physically impossible for isotropic substances, as it would imply that the volume increased under compression, violating conservation of energy. Few experimental determinations of Poisson’s ratio for living plant tissues have been made. They range from 0.18 to 0.4 for onion epidermis [60]-[62]. We find that the growth behaviour of a model is insensitive to the precise value of 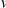 (also see Case 6 in Text S1), and have generally set it equal to 0.3 in our simulations. In our current models, for simplicity we have taken the elasticity properties to be uniform throughout the tissue and over the time of its development. However, elasticity that varies over the tissue and over time can also be described using the GPT-framework.

The analysis so far has assumed that the deformations to be computed are always small. Growth by large amounts can be computed iteratively, by growing in a series of small time steps, in each of which the growth causes only a small deformation. The result is to produce a plastic flow of the material over large time intervals, computed by the theory of small deformations of purely elastic material.

### Relationship between GPT-framework and plant growth

Plant growth is thought to occur from a transient reduction in the stiffness of cell walls allowing them to stretch under turgor pressure, new material being added to restore the stiffness [16], [17], [29], [63]–[67]. When the process is anisotropic, it may be because the cell wall fibres typically have coherent directionality, or because the weakening is distributed non-uniformly over the walls of a cell. Most studies have simplified the process by assuming that it is equivalent to increasing the amount of material in a region and then relaxing the shape. This is also the approach used in the GPT-framework : the *specified-strain* model. The simplification avoids the need to measure relative stiffness and consider turgor pressure. We show below how this approach is related to turgor-based systems.

Suppose the tissue has stiffness tensor field 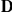, and turgor pressure field 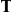. As a result, it must be in some state of strain 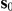. The resulting stress in the tissue is 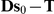. The condition for the tissue to be in equilibrium is that for any small displacement 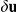, 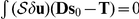. Now suppose we change the rest state. For any small piece of the tissue, its rest state is the shape it would be in if the turgor were removed (and the mechanical linkage to the rest of the tissue ignored). 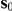 is the transformation from that state to the state that it takes up under turgor. Changing the rest state means applying a strain 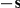. (The minus sign is due to the fact that we want positive values of 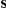 to model an increase in the resting size, but the effect of increasing the resting size is to put the tissue into a state of compression, which is described by negative values of strain.) When turgor is reapplied, the resulting strain is 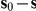. (To validly add strains like this, we are assuming that all of the strains are small.) The applied strain 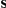 will produce some equilibrium displacement field 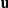. The condition for the new equilibrium is 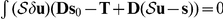. We can subtract from this the original equilibrium condition, leaving 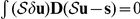, our original equations (2) and (3). This means that the effect of a specified strain field 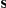 is independent of the turgor, and we can ignore the turgor in our calculations.

The following two theorems explore the relationship between the method of specifying the strain and the method of modulating the stiffness tensor.

**Theorem 1** *Let a tissue have a stiffness tensor field* 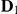, *a turgor pressure field* 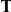 *and a strain field* 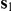, *such that the tissue is in mechanical equilibrium. Suppose that the stiffness field is then changed to* 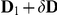, *where* 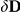 *is small compared with* 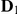.

*Let a second tissue of identical geometry have a stiffness tensor field* 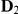 *and be in equilibrium under a strain field* 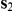.

*Then there is a specified strain tensor field* 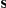 *such that the deformation of the second tissue resulting from applying* 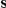 *is the same as the deformation of the first tissue resulting from the change in stiffness* 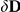.

The strain field 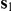 can be split into two parts: the strain due to turgor, which is 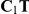 (where 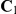 is the compliance tensor, i.e. the inverse of 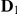), and a residual strain 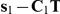. The residual stress field in the first tissue is the residual strain multiplied by 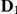, which is 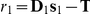. For the tissue to be in equilibrium in this state, the work done by any infinitesimal displacement field 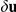 against the residual stress must be zero. This work is 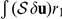 where the integration is over the whole tissue. Recall that 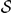 is the differential operator that computes the strain tensor field of a displacement field.

If the stiffness is changed to 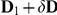, a new equilibrium configuration will be established by a displacement field 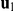. The residual stress field is then 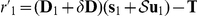, and in equilibrium we have 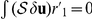 for all 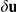. Subtracting the previous virtual work equation gives 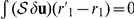. 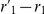 is equal to 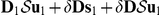. If we assume that 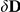 is small in comparison with 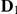 and 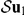 is small in comparison with 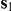, then the last term can be omitted as being of second order, leaving an effective residual strain of 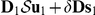.

In the second tissue, the residual stress is initially 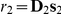, and equilibrium implies that 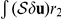 is zero for all virtual displacement fields 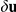. When the strain 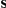 is applied, it will produce a displacement field 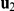, and a residual stress 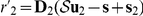. As for the first tissue, to determine the equilibrium value of 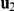 we need only consider the effective residual stress 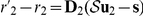.

To prove the theorem it is sufficient to find a value for 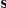 such that when 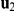 is taken equal to 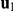, the residual strains 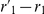 and 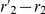 are identical at every point. Thus we require 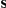 to satisfy:
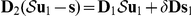



Since 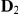 is invertible, its inverse being a compliance tensor 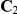, we can immediately calculate 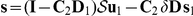, where 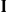 is the identity matrix. This proves the Theorem.

If the first tissue of this theorem is a biologically accurate description of an increment of growth in terms of the tissue’s background stiffness 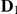, turgor 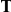, and change in stiffness 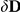, then the theorem tells us that we can find another description in terms of a specified strain 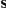 which gives the same deformation. Furthermore, we have a free choice of the background stiffness 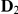. In particular, we can choose 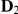 to be uniform and isotropic, and constant over time. However, the relationship between 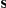 and 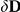 is somewhat complex. When using specified strain to model the result of growth by stiffness modulation, we would like to obtain a closer connection between 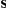 and 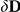, which we now proceed to do.

Firstly, if we take 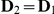, then the expression for 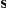 simplifies to 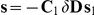, and we need no longer calculate 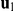.

Now suppose that 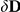 is orthotropic. That is, at each point there are three orthogonal axes such that the change in stiffness is symmetric under a half-turn about each of them. These are called the principal axes of 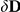. Under certain extra conditions, we find that the principal axes of 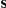 coincide with those of 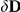. Thus the same distribution of polarisation can be used for either description of growth.

**Theorem 2** *Under the conditions of Theorem 1, suppose that the following conditions hold:*

1. 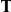, 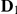, *and* 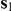 *are everywhere isotropic.*

2. 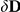 *is orthotropic.*

*Then by taking* 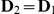, *the principal axes of the specified strain* 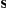 *given by Theorem 1 coincide with those of* 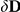.

We have seen already that if 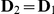, then 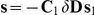. 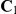 and 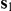 are isotropic by the first condition. By the second condition, 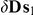 is the stress associated with the isotropic strain 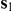 given the change in orthotropic material properties 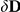. Such a stress has the same principal axes as 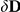. Multiplying by the isotropic compliance 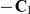 leaves the axes unchanged.

### Implementation

#### Numerical methods

**Figure 12:**
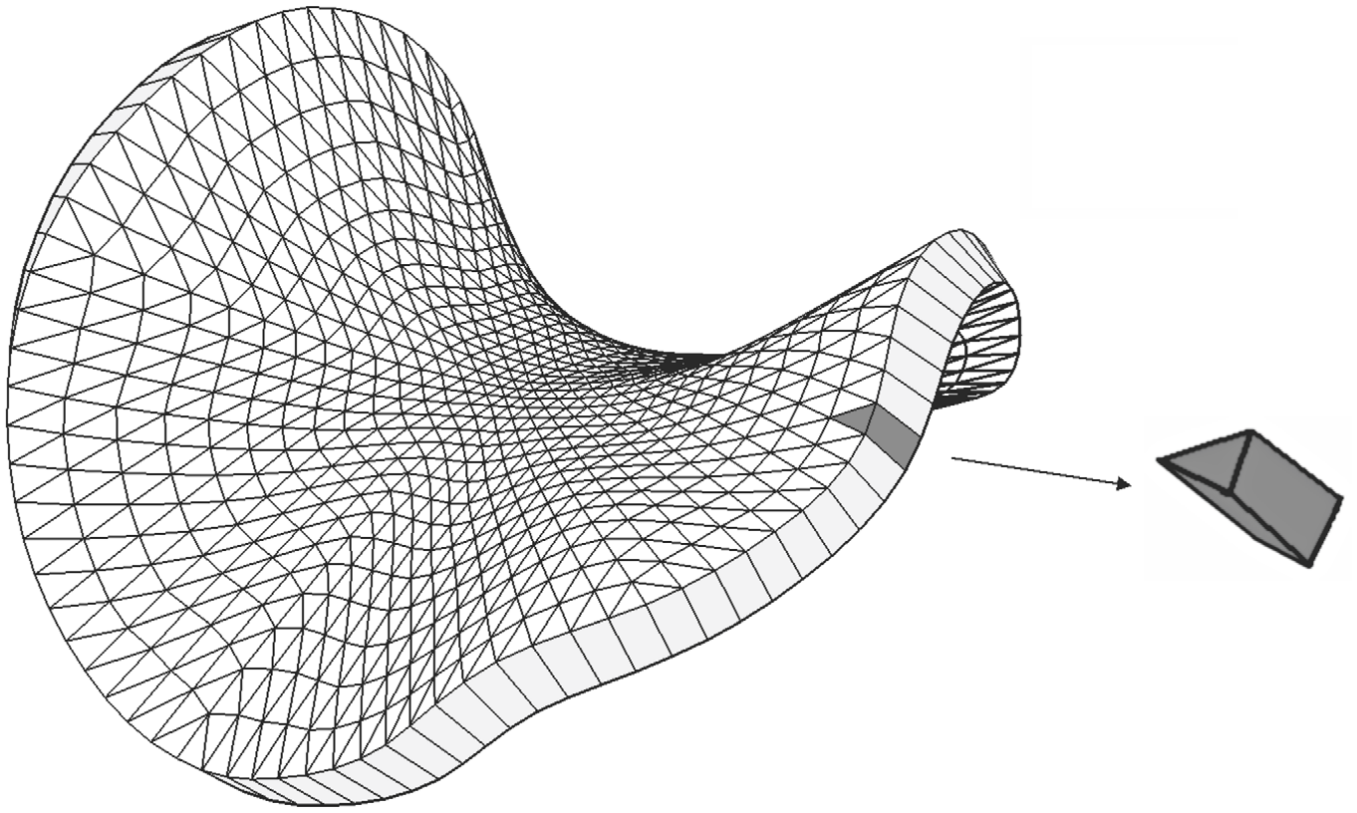
A curved canvas is approximated as a mesh of pentahedra.

We solve the elasticity problem by the finite element method applied to the equations of linear elasticity [55], [56]. The first step is to represent the initial shape of the continuous tissue by a large number of small pieces with simple shapes. These are the “finite elements” for which the method is named. For the particular tissues we examine in this paper, which are of finite but small thickness, we divide the material into a mesh of pentahedra. These resemble triangular prisms, except that they need not be regular and their quadrangular faces need not even be flat. Their opposite triangular faces lie on the two sides of the canvas, and divide them into isomorphic triangular meshes. An example of a discretised canvas is shown in Figure 12.

For accuracy and stability of the computations, no finite element should be excessively longer in any direction than it is in any other: a ratio of up to 10 is practical. In particular, its diameter along the surface should not be excessively greater than the thickness of the surface. For extremely thin surfaces, this would necessitate excessively large numbers of finite elements, and for such surfaces, the methods of thin shells are to be preferred, which model the surface as being a substance of zero thickness with intrinsic bending stiffness properties. However, for the tissues we are concerned with, the thickness is large enough for three-dimensional elements to be practical, while also small enough that a single layer of them suffices.

All continuous properties of the tissue–morphogen concentrations, growth tensors, displacements, and the position of the tissue itself–are represented by the values they take at the vertices of the finite element mesh, and are interpolated over the interior of each element. We adopt the notation that for any property 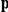 defined at every point of the continuous tissue, the vector consisting of all the values it takes at the vertices (in some arbitrary but fixed order) is denoted by 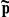. Provided that 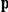 does not vary very much over a single finite element, the interpolation of 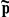 will be a good approximation to the original field 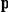.

In a small time step 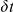, the growth rate field gives a field of specified growth, which determines a deformation field by Equation (3). If in this equation we replace the unknown deformation field 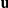 and the known specified growth tensor field 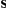 by their interpolated approximations and perform the integration numerically, we obtain a set of linear simultaneous equations in the unknown vertex displacements. We defer details to Text S1. The equations have the following general form.
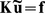
(4)Here 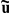 is the concatenation of all of the unknown vertex displacement vectors. 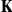 is a square matrix computed from the geometry and elasticity properties of the object, and 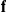 is computed from these properties together with the specified growth field.

Since we are assuming linear elasticity, the time step, and hence the specified growth, should be chosen small enough for the linear approximation to be accurate. A rule of thumb is to take the time step to be small enough that the amount of specified growth in that time interval is nowhere greater than 10%, and the resultant rotations are nowhere more than 10 degrees. A validity check can be made by re-running a simulation with smaller time steps and confirming that the same results are obtained.

The residual growth field 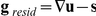 can be numerically obtained from 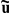 by interpolating the displacements over the finite elements and differentiating to obtain an approximation to 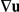. The residual strain field and the rotation field are obtained as the symmetrical and skew-symmetrical components of the residual growth: 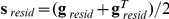 and 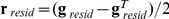.

If some parts of the substance are required to remain stationary, this amounts to stipulating that certain components of 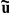 be zero. The corresponding components of 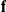 will then have additional unknown terms added to them for the unknown forces that must be present to keep those components stationary. Thus where components of 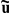 are known in advance, the corresponding components of 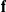 become unknowns. If we do not want to compute the unknown forces, then we can simply eliminate the relevant equations. Such conditions can be applied to individual coordinates, for example, holding the 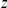 coordinate of a vertex fixed while allowing it to move in the 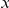 and 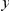 directions. This is done for all of the examples of Figure 3, where every vertex of every finite element on the boundary of the tissue is constrained to not move in the vertical direction, to simulate the presence of an extended tissue around the part that is being simulated and shown in the figure. Problems in which the canvas is required to remain flat but its thickness is allowed to vary can be handled by adding the constraint that 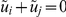 whenever 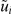 and 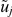 are the 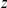 components of the displacements of corresponding points on the two sides of the canvas. 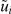, 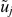, and the unknown forces which are implied by the constraint, can then be eliminated from the equations and replaced by 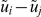, the thickness at that point.

#### Software for modelling in the GPT-framework

*GFtbox* is implemented in MATLAB, which provides easily programmed access to fast array-handling routines. Building the array 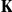 and solving equation (4) takes most of the computing time. 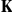 is an 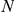 by 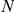 matrix, where 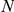 is the number of spatial degrees of freedom: three times the number of vertices in the mesh. The number of finite elements will be approximately the same as the number of vertices. The size of problem that can be handled is limited primarily by the amount of memory available. Currently we are able to run simulations with up to about 5,000 finite elements. The number of nonzero elements of 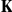 is proportional to 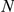, and so most elements of 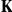 are zero. This allows “sparse” representations of the matrix, which save space by storing only the non-zero elements.

The simultaneous linear equations (4) are solved for 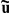 using the conjugate gradient squared method, available in MATLAB as the function cgs. Other methods of solution exist, but we have found this to be the most robust and efficient, and one which works well with sparse matrices. Although 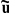 is only determined up to a rigid motion of the whole mesh (and therefore 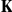 is always rank deficient by at least 6), in practice the cgs method finds a solution without introducing random drift.

The accuracy of the computations and the time that they take depend on several factors: the tolerance with which equation (4) is solved, the time step, the fineness of the division into finite elements, and the quality of the elements. The examples in this paper were generally computed with between 3000 and 5000 finite elements, and where necessary, dynamic remeshing was used to maintain their quality. The tolerance for solving equation (4) was typically set to 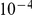 or less for elasticity, and 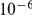 for diffusion. (The tolerance is interpreted relative to the magnitude of the numbers in the equations, and can optionally be measured either in terms of the root mean square of the errors or their maximum absolute value.) The computation from initial to final states was divided into between 50 and 200 time steps. For those examples where an analytic solution can be found, the computation agreed to within a fraction of a percent. Several such validation tests are described in Text S1. Time per iteration is roughly proportional to the number of elements, and was around 15 seconds to 1 minute (equivalently, between 5 and 20 ms per finite element).

*GFtbox* supports dynamic remeshing. The need for this arises when a canvas grows anisotropically and the finite elements become long and thin. This tends to make the finite element equations ill-conditioned. In addition, whatever their shape, if the diameter of the finite elements becomes significant relative to the scale on which the mesh is bending or non-uniformly deforming, they will produce an artefactual stiffness. It may therefore be necessary to adapt the mesh as it grows, to maintain both its numerical quality and its fit to the continuous canvas that it models [68]. This is done in two ways: edges of the mesh (and the finite elements on either side) are split when they exceed a certain length, and local transformations are performed to eliminate thin elements.

For this purpose we consider the triangular mesh formed by the midplanes of the pentahedra. In general, the quality of the mesh is better maintained if many edges are split at once than if they are split one by one. Therefore there are two thresholds for splitting: whenever any edge exceeds the upper threshold, every edge exceeding the lower threshold is split. The new vertices could be placed simply by bisecting the edges to be split. However, this would simply subdivide each finite element into a flat mesh of smaller elements. Since the flatness of each finite element is to be considered as an approximation to a smoothly curved surface passing through all the vertices, it is preferable to place the new vertices so that successive subdivisions yield such a surface. This can be done by the butterfly subdivision rule [69], which places the new vertex at a certain weighted average of vertices in the neighbourhood of the split edge that include more than just the two endpoints. Each new vertex of the triangular mesh requires two pentahedron vertices to be created, one on each side of the canvas. These are found by applying the same butterfly interpolation to the corresponding vertices on the two sides. The values of growth factors and signals at the new vertices are interpolated in the same way.

Growth can often be continued indefinitely in this manner without loss of quality of the triangulation, and is limited only by the total number of finite elements that can be handled.

### Specifying the strain tensor by growth factors

We now turn to how the specified growth tensor field 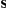 is determined by concentration fields of growth factors, and how such concentration fields can be created by defining methods of production, consumption, diffusion, and interaction.

### Determining the specified growth tensor

A specified growth tensor has three principal axes at right angles to each other. When the tissue is a curved canvas of finite thickness, we assume that although the two sides of the canvas may grow at different rates, they have the same directions of principal growth axes, one of which is always perpendicular to the mid-plane of the canvas, the other two being parallel to it. These axes and the corresponding growth rates are determined by concentration fields of factors. We assume that factor concentrations do not vary through the thickness of the tissue, and therefore represent them computationally by their values on a two-dimensional mesh of triangles, being the midplanes of the mesh of pentahedra used for the elasticity calculation.

In the GPT-framework factors can be classified into two types. *Identity* factors do not propagate within the canvas, while *signalling* factors can. The specified growth tensor at each point of the canvas is parameterised as follows. The specified principal directions of growth within the plane of the canvas are determined by the gradient of a signalling factor called *POL*. The specified rates of growth parallel to these directions on the two surfaces of the canvas are given by factors called 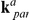 and 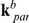. Likewise, the specified growth rates perpendicular to the polarising gradient 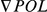 are given by factors 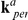 and 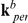. The rate of growth of thickness of the canvas is specified by a factor 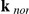.

### Propagation

Propagation of signalling factors may occur through a variety of mechanisms, such as diffusion or active transport. Here we implement diffusion which biologically may be a proxy for a variety of underlying mechanisms. The evolution of a concentration field 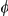 is modelled by the following equation:
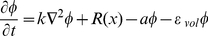
(5)The four terms on the right hand side represent respectively diffusion (with a diffusion constant 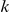), production at a rate 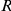, decay at a uniform rate 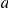, and the diluting effect of growth. Here 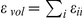 is the volumetric rate of resultant growth. Our implementation handles dilution as a separate step and does not include it in the equation (see later). The values of 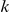, 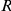, and 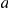 may vary in space. They may also vary in time, but we assume not rapidly, so that they can be assumed constant over a single timestep.

To obtain the concentration field at time 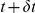, we can make a linear approximation and write a forward Euler equation:
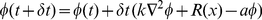
(6)


By methods similar to those for elasticity, we can discretise this relationship, representing the concentration distribution by its values at the vertices, and obtain an equation similar in form to equation (4): 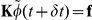. 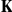 and 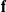 are calculated from the geometry of the mesh, the diffusion constant, the production and decay rates, and the current distribution 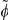. This set of equations can be solved to give the new concentration distribution. As for the calculation of displacements, the equations allow boundary conditions to be added stipulating that the concentration remains fixed at some nodes.

Unlike the case of elasticity, here 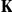 has full rank and the solution is uniquely determined. The sizes of 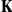 and 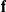 are 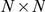 and 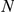 respectively, where 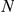 is the number of vertices of the triangular mesh. This is 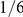 of the value of 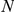 for the elasticity computation, resulting in a much faster solution. A comparison of a computed diffusion pattern with its analytical solution is considered in Text S1. In the case of morphogens which do not diffuse, it is not necessary to solve the diffusion equation, and the effects of production and decay can be calculated directly, vertex by vertex.

We compute diffusion separately from elastic deformation. In principle, the diffusion problem could be solved for a growing and deforming canvas, but over a short time interval only second-order effects arise from the interaction between growth and diffusion, except for the dilution effect mentioned above. When a material expands, the concentration of a physical substance spread through it must decrease in proportion. We make this correction as a separate step: after the diffusion and elasticity calculations, the concentration for each factor subject to dilution by growth is reduced at each point by the proportional expansion at that point, 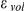.

It is unrealistic to assume that cells can detect the directions of arbitrarily shallow gradients; these also pose numerical problems. There are various options for dealing with very shallow gradients. (1) Generate new sources or sinks for signalling factors as space is created through growth. This would enable patterns to be continually elaborated as the shape expands. (2) Fix the pattern before it becomes too shallow. In the particular case of tissue polarity, for example, polarity may be frozen when the magnitude of the POL gradient falls below a certain threshold. This would be equivalent to a cell becoming polarised when the tissue is small enough for gradients to be measurable, and then retaining its polarity when the gradient falls below the threshold of detectability. Alternatively, (3) the polarity can disappear, resulting in isotropic growth.

Case Z: Partitioning a canvas using a diffusing signal.

**Figure 13:**
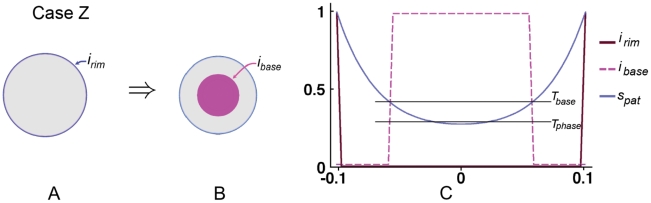
Patterning in the canvas. (A) The initial disc has a pre-defined region at the rim, 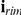 (blue). (B) The inner region, 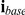 (magenta), is obtained through a combination of diffusion and interaction of a signal (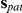) produced by 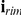. (C) Profiles of the factors (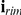, 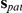, 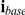) plotted along a diameter together with the thresholds, 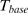 and 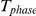.

Figure 13 shows a hypothetical example of how diffusion and thresholds can lead to the canvas being partitioned into regions to create a new central region from a peripheral one. An identity factor 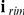 is expressed at the rim of the disc-shaped canvas (blue in Figure 13 A,C). The expression of an identity factor is represented by the value 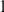 and non-expression by the value 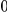. (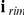 could represent a transcription factor expressed only at the rim.) Initially a signalling factor 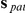 (blue in Figure 13 C) is present everywhere (in this model an initial value of 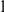) and its rate of production is promoted by 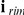. Diffusion and decay cause the level of 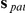 to drop in the centre until it reaches a steady state, bowl shape, as shown in Figure 13 C. Wherever 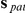 drops below a threshold 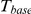 the identity factor 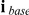 is expressed (is set to the value 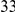) so defining a new central region. In this example, 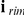 can be considered as a regional organiser as it provides a source of the signalling factor, 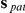, that enables regions to be elaborated.

This patterning process could also be used to control the timing of particular events. For example, there can be a further factor, 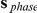 with a high propagation rate, which is generated where 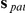 falls below a second threshold 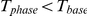. When this threshold is reached, 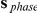 will propagate rapidly to activate or inhibit factors bringing in a new patterning phase.

### Interaction between factors

Identity, signalling, and growth parameters may interact in many different ways. Rather than assume a fixed set of possible interactions, the software allows the user to write a general function to model interactions, the “interaction function”. The function is called on every iteration of the simulation, before the calculation of diffusion and growth. To simplify the task, a few standard functions are provided to model promotion and inhibition of one factor by another. These are:
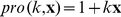
(7)

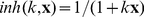
(8)


We use boldface for vectors of values, one value per mesh vertex, and italic for scalar values. Multiplication and division of vectors are to be understood elementwise. 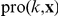 and 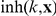 both tend to vectors of 1’s as the components of 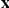 tend to zero. If factor 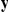 is to be assigned the value of factor 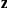 promoted by factor 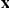 by an amount 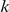, one writes the MATLAB equivalent of 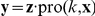 (i.e. y = z.*pro(k,x);). If 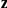 is to be inhibited by 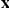, then 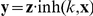. It is convenient to express inhibition and promotion in this way because the overall effects of different factors (say 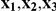) that may be expressed in different regions can be obtained by multiplication (e.g. 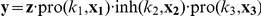).

### The complete simulation loop

The iterative loop of the simulation combines the regulatory and mechanical systems as follows.

1. Calculate the levels of growth factors from their interactions. This is usually specified in the interaction function and this step is where hypotheses can be formulated.
2. Calculate the result of diffusion over a small time step 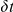.
3. Calculate the specified growth tensor field arising from the factors.
4. Calculate the resulting displacement of every vertex (Equation 4) and update the vertex positions accordingly, from which computed growth field can be obtained.
5. From the displacement field, calculate the field of volumetric growth, and reduce the concentration of every dilutable signalling morphogen in proportion. Where regions of identity factors enlarge, the new volume inherits the identity profile of its parent volume.
6. Optionally, discard or retain part or all of the residual strains resulting from the growth and deformation.
7. Advance simulated time by 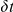.
8. Display the object on screen, save a screenshot, or save a frame to a movie file.

## Supporting Information

Text S1 Illustrative examples of interaction functions, further details of mathematical methods and implementation, and a series of examples of *GFtbox* computations validating the method and the software.
(PDF)

Video S1 A movie of the growth illustrated in Figure 5.
(MOV)

Video S2 A movie of the growth illustrated in Figure 6 (C).
(MOV)

Video S3 A movie of the growth illustrated in Figure 6 (D–E).
(MOV)

Video S4 A movie of the growth illustrated in Figure 7 (B).
(MOV)

Video S5 A movie of the growth illustrated in Figure 7 (C).
(MOV)

Video S6 A movie of the growth illustrated in Figure 7 (E).
(MOV)

Video S7 A movie of the growth illustrated in Figure 7 (F).
(MOV)

Video S8 A movie of the growth illustrated in Figure 21 in Text S1.
(MOV)
