# Supplementary material for: Generation of Diverse Biological Forms through Combinatorial Interactions between Tissue Polarity and Growth
Source: PLoS Comput Biol. 2011 Jun 16;7(6):e1002071. doi: 10.1371/journal.pcbi.1002071 (PMC3116900; doi:10.1371/journal.pcbi.1002071)
Supplement: Text S1 — Illustrative examples of interaction functions, further details of mathematical methods and implementation, and a series of examples of GFtbox computations validating the method and the software. (PDF) [file pcbi.1002071.s001.pdf]

Supplement

In this supplement we provide illustrative examples of interaction functions, further details of mathematical methods and implementation, and a series of examples of *GFtbox* computations validating the method and the software.

Interaction functions

Models can be developed using the *GFtbox* user interface. However, it is desirable to specify the model programmatically. This is done in the "interaction function". On creating a project *GFtbox* automatically generates a prototype interaction function with comments that provide prompts. A typical interaction function is divided into several parts:

1. The setup provides an initial canvas together with a pattern of factors. The initial canvas is imported as a pre-defined mesh of finite elements. The pattern of identity factors is imposed according to the geometry of the mesh. Typically, the values of identity factors range from 0 (inactive) to 1 (fully active).
2. The Polariser Regulatory Network (PRN) specifies the orientation of growth through the action of organisers and background generation and decay rates for the polariser, POL.
3. The Gene Regulatory Network (GRN) details general interactions between factors corresponding to genes.
4. The Growth Regulatory Network (KRN) details the interactions between factors as they specify growth rates, $\mathbf{k}_{par}, \mathbf{k}_{per}, \mathbf{k}_{nor}$.

Outlines of the interaction functions for three cases (Cases R, S and T) follow.

Case R, outline of interaction function

Setup The initial canvas 0.8mm in diameter is imported complete with two regional factors with values $[0, 1]$, $\mathbf{i}_{upper}$ and $\mathbf{i}_{lat}$ (Figure 5A) and a signalling factor with the range $[0 \ldots 1]$, $\mathbf{s}_{lat}$. The $\mathbf{s}_{lat}$ is generated in the region $\mathbf{i}_{lat}$, has a diffusion constant of $D_{lat} = 0.005\ mm^2/hr$ and decay rate of $a_{lat} = 0.1/hr$. It diffuses out from $\mathbf{s}_{lat}$ region forming an exponential gradient (except in the lower region, $\mathbf{i}_{lower}$).

$$\frac{\partial \mathbf{s}_{lat}}{\partial t} = D_{lat} \nabla^2 \mathbf{s}_{lat} + \mathbf{i}_{lat} \cdot \mathrm{inh}(100, \mathbf{i}_{lower}) - a_{lat}\, \mathbf{s}_{lat}$$

PRN None.

GRN

$$\mathbf{i}_G = 0.016\, \mathbf{i}_{upper} \cdot \mathrm{inh}(7, \mathbf{s}_{lat} \cdot \mathbf{s}_{lat})$$

KRN

$$\begin{aligned} \mathbf{k}_{par} &= 0.5\, \mathbf{i}_G \\ \mathbf{k}_{per} &= \mathbf{k}_{par} \\ \mathbf{k}_{nor} &= 0.003 \end{aligned}$$

Case S, outline of interaction function

Setup As for Case R and with regionalising factors. $\mathbf{i}_{dist}$ along the rim and $\mathbf{i}_{prox}$ which circles the bottom of the cylinder (Figure 5A)

PRN The polariser is free to diffuse with diffusion constant of $D_{pol} = 0.05\ mm^2/hr$ and decay rate $a_{pol} = 0/hr$ under the influence of organisers:

$$\begin{aligned} \mathbf{i}_{+org} &= \mathbf{i}_{prox} \\ \mathbf{i}_{\text{-}org} &= \mathbf{i}_{dist} \end{aligned}$$

the (+)organiser and -organiser respectively (Figure 6). The organisers specify the gradient of polariser by setting the POL level to the value one (+organiser) and zero (−organiser). These regions of the polariser are then clamped (they will not change, in biological terms they are effectively under homeostatic control). They provide boundary conditions for

$$\frac{\partial POL}{\partial t} = D_{pol} \nabla^2 POL$$

which in the steady state produces an approximately linear gradient.

GRN

$$\mathbf{i}_G = 0.016\, \mathbf{i}_{upper} \cdot \text{inh}(7, \mathbf{s}_{lat} \cdot \mathbf{s}_{lat})$$

KRN

$$\begin{aligned} \mathbf{k}_{par} &= 0.75\, \mathbf{i}_G \\ \mathbf{k}_{per} &= 0.25\, \mathbf{i}_G \\ \mathbf{k}_{nor} &= 0.003 \end{aligned}$$

Case T, outline of interaction function

Setup As for Case S with addition of $[0, 1]$ factors, $\mathbf{i}_{leftline}$ and $\mathbf{i}_{rightline}$ that define proximodistal line regions at 90° to $\mathbf{i}_{lat}$. In addition, $[0, 1]$ factors $\mathbf{i}_{early}$ and $\mathbf{i}_{late}$ where $\mathbf{i}_{early} = 1$ until 120 hrs. and $\mathbf{i}_{late} = 1$ subsequently.

PRN As for Case S except

$$\mathbf{i}_{distorg} = \mathbf{i}_{early} \cdot \mathbf{i}_{dist} + \mathbf{i}_{late} \cdot (\mathbf{i}_{leftline} + \mathbf{i}_{rightline})$$

GRN As for Case S.

KRN As for Case S.

Further details of numerical methods

The heart of the numerical implementation of the finite element method is the computation of the matrix $\mathbf{K}$ and the vector $\mathbf{f}$ used in equation (4) to compute the elastic deformation, and the equation of similar form for numerical solution of the diffusion equation. Here we give a necessarily brief outline of the general method as applied to the elasticity problem. The discussion is based on [56] vol.1, chs.1–2. (Citations refer to the bibliography in the main paper.)

We first consider a single finite element in isolation. Its vertices are $x_i, i = 1 \ldots 6$. The morphogens determine a specified strain field over the element. This field is computationally represented by its values s_i at the six vertices, and are interpolated over the whole element by a set of functions called *shape functions* ([56], ch. 2). These functions specify how the value of $\mathbf{p}$ at any point in the interior of a finite element is approximated by a weighted average of the values of $\tilde{\mathbf{p}}$ at the vertices of the element. For each finite element there is a shape function N_i for each vertex v_i of that element. It maps every point of the finite element to a real number between 0 and 1. It takes the value of 1 at v_i, is zero at every other vertex, and varies continuously everywhere else. The sum of all the shape functions is 1 everywhere within the element. Outside the element they are all zero. If p_i is the value of $\mathbf{p}$ at v_i, the interpolated value at any point $\mathbf{v}$ in the element is $\sum_{i=1\ldots6} N_i(\mathbf{v})p_i$.

The shape functions that we use are most simply expressed in terms of a standard pentahedron, whose six vertices we denote by $\mathbf{v}_i^s$, $i : 1 \ldots 6$. These are $\mathbf{v}_1^s = (1, 0, 1)$, $\mathbf{v}_2^s = (0, 1, 1)$, and $\mathbf{v}_3^s = (0, 0, 1)$, and $\mathbf{v}_{4\ldots6}^s$ which are the same with a z coordinate of -1. These form a triangular prism. Within this prism, the six shape functions are defined by $N_1^s(x, y, z) = x(1 + z)/2$, $N_2^s(x, y, z) = y(1 + z)/2$, and $N_3^s(x, y, z) = (1 - x - y)(1 + z)/2$, and $N_{4\ldots6}^s$ the same replacing z by $-z$. Outside the prism they are all zero. Now let $\mathbf{v}_{1\ldots6}$ be the vertices of a general pentahedron. The shape functions for this pentahedron will be defined by a transformation from the standard pentahedron. Let $\mathbf{u}^s$ be any point in the standard pentahedron. The standard shape functions N_i^s are designed so that $\mathbf{u}^s = \sum_{i=1\ldots6} N_i^s(\mathbf{u}^s)v_i^s$. Let $\mathbf{u} = \sum_{i=1\ldots6} N_i^s(\mathbf{u}^s)v_i$. The range of this mapping from $\mathbf{u}^s$ to $\mathbf{u}$ defines the shape of the general pentahedron on the vertices $\mathbf{v}_{1\ldots6}$. Its shape functions are defined on that volume by $N_i(\mathbf{u}) = N_i^s(\mathbf{u}^s)$. Outside that volume, they are defined to be zero.

Besides defining the method of interpolation, the shape functions also define the space occupied by the element (hence their name): it is the set of points for which at least one of the shape functions is positive. The surface of the element is the subset of the volume for which at least one shape function is zero. For the triangular faces these are flat triangles. For the quadrangular faces these are in general curvilinear surfaces. The shape functions have been defined in such a way that on each face, the shape functions are independent of the positions of the vertices not lying on that face. This ensures that when adjacent finite elements share a quadrangular face, they fit together without voids, forming a continuous solid. Furthermore, when any quantity is interpolated over all the finite elements by means of the shape functions within each element, the resulting field is continuous.

For the specified strain field, we thus have:

$$\mathbf{s}(x) = \sum_{k:1\ldots6} N_k(x)\mathbf{s}_k$$

The displacement field that we want to compute is represented by the displacements of the six vertices, which by interpolation define the displacement of every point of the element. This (still unknown) displacement field has a spatial gradient field, of which we can extract the strain component. These can

be expressed by interpolation using the gradients of the shape functions:

$$\begin{aligned}\mathbf{u}(x) &= \sum_{k:1\ldots6} N_k(x)\mathbf{u}_k \\ \nabla\mathbf{u}(x) &= \sum_{k:1\ldots6} (\nabla N_k)(x)\mathbf{u}_k \\ (\mathcal{S}u)(x) &= \sum_{k:1\ldots6} (\mathcal{S}N_k)(x)\mathbf{u}_k\end{aligned}$$

where $\mathcal{S}$ is the differential operator $(\mathcal{S}\mathbf{u})_{ij} = (\frac{\partial u_i}{\partial x_j} + \frac{\partial u_j}{\partial x_i})/2$. A general treatment of elasticity problems would take into account external applied forces, but these are absent from our present computations.

For computational purposes, any symmetric tensor such as the strain tensor ε can be represented as a vector of its six independent elements $(\varepsilon_{xx}, \varepsilon_{yy}, \varepsilon_{zz}, \varepsilon_{yz}, \varepsilon_{zx}, \varepsilon_{xy})$. With respect to this representation, the $3 \times 3 \times 3 \times 3$ elasticity tensor $\mathbf{D}$ can be represented by a 6×6 symmetric matrix which operates on these strain vectors by matrix multiplication. This representation allows equation (2) to be written in matrix notation as

$$W(\delta\mathbf{u}) = \int \sum_{k:1\ldots6} (\mathbf{B}_k(\delta\mathbf{u})_k)^T \, \mathbf{D} \, (\mathbf{B}_k\mathbf{u}_k - \mathbf{s}_k)$$

where $\mathbf{B}_k = \mathcal{S}N_k$. For this to be zero for all $\delta\mathbf{u}$, we must have

$$\int \sum_{k:1\ldots6} \mathbf{B}_k^T \, \mathbf{D} \, \mathbf{B}_k\mathbf{u}_k = \int \sum_{k:1\ldots6} \mathbf{B}_k^T \, \mathbf{D} \, \mathbf{s}_k$$

If we now let $\tilde{\mathbf{u}}$ be the concatenation of the vectors $\mathbf{u}_k$, and similarly combine the matrices $\mathbf{B}_k$ into a single 6×18 matrix $\mathbf{B}$, then the equation takes the form

$$\textstyle\int \mathbf{B}^T\mathbf{D}\mathbf{B}\tilde{\mathbf{u}} = \int \mathbf{B}^T\mathbf{D}\,\mathbf{s} \qquad \text{(S1)}$$

As $\mathbf{B}$, $\mathbf{D}$ and $\mathbf{s}$ can be evaluated at any point within the finite element, it is possible to integrate $\mathbf{B}^T\mathbf{D}\mathbf{B}$ and $\mathbf{B}^T\mathbf{D}\mathbf{s}$ exactly, but in practice it is faster to numerically approximate the integrals by a weighted sum of their values at carefully chosen points within the element (a method called Gaussian quadrature). The result is equation (4) for a single finite element: $\mathbf{K}\tilde{\mathbf{u}} = \mathbf{f}$. Note that in equation (S1), the elasticity tensor appears on both sides. When the elasticity tensor is isotropic and expressed in terms of the bulk modulus and Poisson's ratio, every element is proportional to the bulk modulus. The bulk modulus therefore cancels out of the equation. This would not be the case if we wished to handle external applied forces; alternatively, such forces can be scaled by the bulk modulus.

Finally, to combine all of the finite elements together, suppose that the above calculation is for the nth finite element, and rename the quantities as $\mathbf{K}_n$, $\tilde{\mathbf{u}}_n$, and $\mathbf{f}_n$. Let $\tilde{\mathbf{u}}$ be the concatenation of the displacement vectors of all the vertices of the mesh. Each finite element will use some set of six vertices, defining a mapping of the indices $1 \ldots 18$ of $\tilde{\mathbf{u}}_n$ to 18 indices of the total vector $\tilde{\mathbf{u}}$. The matrix $\mathbf{K}_n$ similarly maps to an 18×18 (non-contiguous) submatrix of a total matrix $\mathbf{K}$, and $\mathbf{f}_n$ maps into $\mathbf{f}$. The result is equation (4) for the whole mesh. Where vertices are shared among elements, overlapping elements of the matrices $\mathbf{K}_n$ and of the vectors $\mathbf{f}_n$ are added together. Note that $\mathbf{K}$ depends only on the elasticity properties and the geometry of the mesh, while $\mathbf{f}$ depends also on the growth.

For diffusion we can derive an equation of a similar form from the continuous diffusion equation and the triangular mesh of midplanes of the finite elements. When there are N vertexes in the triangular mesh there are N degrees of freedom for the diffusion problem, instead of $6N$ for elasticity, so solution is much faster. We could take advantage of this (but have not yet done so) by solving the diffusion equation on a finer mesh than for elasticity, using values of the morphogens not only at the vertices but also at the edge midpoints.

Validation tests

In the following examples the true solution can be calculated and compared with the computed solution. In many examples a grid is drawn on the surface, dividing it into squares in the initial state, to make local growth and three-dimensional shape more visible. These squares are not the finite elements: the computations were carried out with each square typically divided into 32 triangular elements, giving a total of 3200 elements for the whole tissue.

Case 1: Rotational invariance. If an arbitrary rotation is applied to the initial mesh, the solver should give the same solution, similarly rotated. Taking the initial mesh of Figure 14A, we compute its development (i) for a fixed length of time without rotations (e.g. Figure 14B) and (ii) first rotating the mesh, then computing its development for the same length of time, then applying the inverse rotation. The results (not shown) are indistinguishable by eye. The magnitude of the errors depends on the tolerance with which equation (4) is solved and the size of the time step.

Case 2: Invariance with respect to the mesh. The same results should be obtained, up to the expected numerical error, regardless of how the surface is divided into elements, provided that they are neither too large nor excessively long and thin. Several variants of a single shape were computed, in which the initial finite element mesh was varied, while leaving the overall size and shape unchanged. The mesh was grown in three dimensions, with the fixed small initial deformation out of the plane. Figure 14 shows the result of growing each of these examples for the same length of time, and picturing the result from the same viewpoint.

(a) The original version divides each of the grid squares drawn in the figures into a 5 by 5 subgrid, each square of which is divided diagonally into two triangular finite elements. The chosen diagonals alternate in adjacent squares.

(b) All the diagonal divisions were in the same direction, to demonstrate that this does not bias the development.

(c) Each large grid square was divided into 3 by 3 smaller squares, each divided diagonally.

(d) The subdivision of the mesh varied in fineness. In the figure, the front edge is divided into 50 elements, and the rear into 20, the size of the triangles varying continuously from front to back.

(e) Each large grid square was divided into 5 rectangles by 2, each diagonally split.

The differences are slight, and are probably mainly due to the fact that the mesh starts nearly flat, and its initial buckling out of the plane is sensitive to the initial conditions.

Case 3: Invariance with respect to the time step. Results will be inaccurate if the time step is too large. A test for whether the time step is small enough is to run the simulation again with a smaller time step and see if the result is substantially different. Figure 15 shows the result of several computations of the same example as in Figure 14. In the first, two time steps are spent on the initial phase of diffusion without growth, and two further steps for the growth. In the second, five steps for each phase, and thereafter each computation uses twice as many steps of half the size as the previous. Significant differences of shape can be seen among the first three, but the remainder are almost indistinguishable.

Case 4: Diffusion from a point or line source. When there is no production, absorption, or growth, the diffusion equation 5 simplifes to the Laplace equation $\partial f/\partial t = D\,\partial^2 f/\partial t^2$, where D is the diffusion constant. For biochemicals it is convenient to measure this in units of $\mathrm{mm}^2/\mathrm{hr}$.

If we place a unit amount of a diffusible substance at a single point, then it will diffuse in a circularly symmetrical manner to yield, at time t, a Gaussian distribution described by $f = A\exp(-r^2/2\sigma^2)$, where f is the concentration, r is the distance from the point, and σ is the standard deviation of the distribution, equal to $\sqrt{2Dt}$.

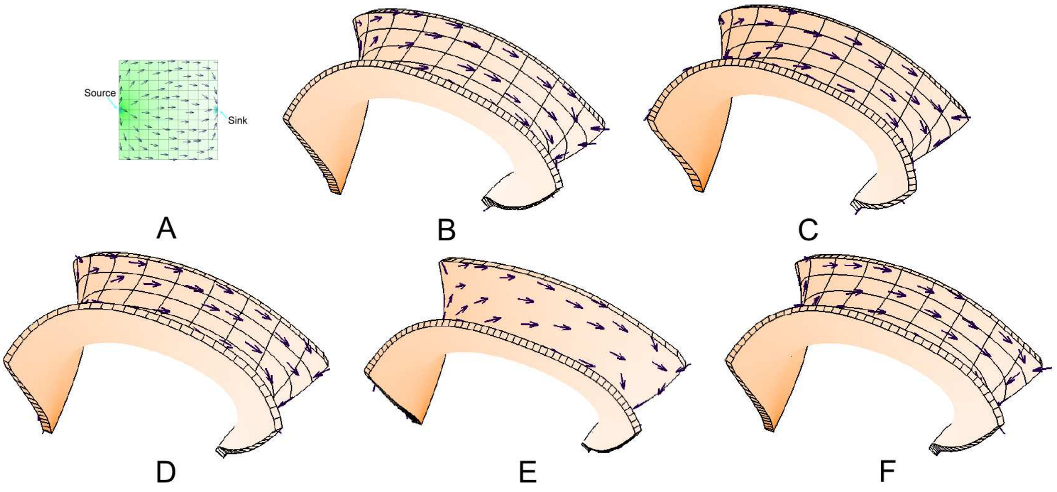

Figure 14. Case 2. Demonstration of invariance with respect to the finite element mesh. (A) Initial square mesh divided into 50 by 50 pairs of triangular elements. (B) The result of growing to time t. (C) The same with all 50 by 50 squares divided along the same diagonal. (D) Division into 30 by 30 pairs of triangles. (E) Divided into 50 elements along each of three edges, 20 along the fourth (the rear edge). (F) Division into 50 by 20 pairs of triangles.

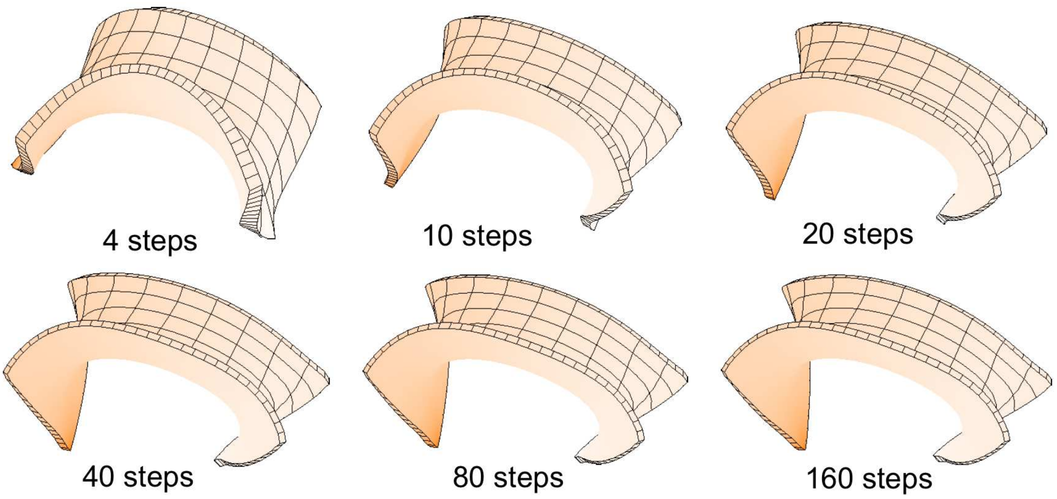

Figure 15. Case 3 Demonstration of invariance with respect to the time step. All simulations ran for the same length of simulated time, divided into different numbers of steps. In the first two runs, the time step is too large for good accuracy. The last three runs are hardly distinguishable by eye.

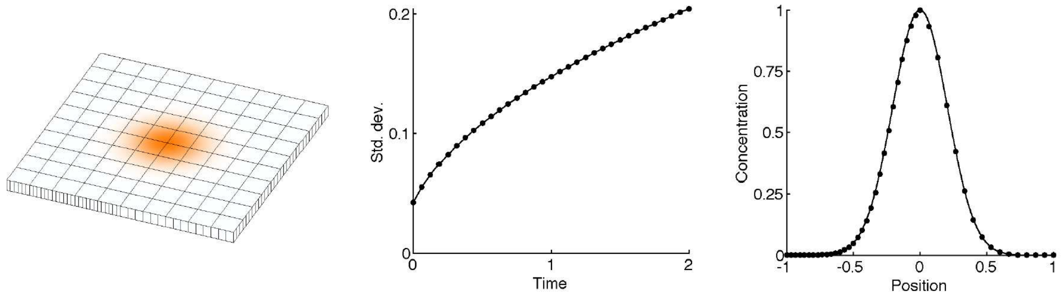

A B C

Figure 16. Case 4 A small source diffuses to give a Gaussian distribution. Mesh diameter = 2, diffusion constant = 0.01, time = 2. (A) Distribution visualised by colour intensity. (B) Computed (dots) and theoretical (line) standard deviation plotted against time. (C) Computed (dots) and theoretical (line) values through a cross-section at time 2.

In the finite element model, if the initial distribution of the substance is smaller than the size of a finite element, then the computation cannot be expected to be accurate, at least until it has diffused outwards to cover an area of several elements. For Figure 16, the substance was initially placed at four vertices near the centre of the mesh. Panel (A) shows the distribution after two time units. In Panel (B) the theoretical (solid line) and computed (dots) standard deviation of the distribution are shown as a function of time. Panel (C) shows the theoretical values (solid line) and the computed values (dots) along a cross-section through the centre at time 2, normalised to the value at the peak. The computed values are spaced twice as closely on the left half of the graph; this is because the surface is divided twice as finely into finite elements on that side, to demonstrate that the computation is independent of the division into finite elements. Even although the initial distribution covers only a few finite elements, the computation agrees to high accuracy throughout with the theoretical prediction for a continuous medium.

A further check is to calculate the total quantity of the substance. If it diffuses without being produced or decaying, then it must be a conserved quantity. For this example, the total quantity does not vary by more than a few parts in 10^{-6}.

Similar results are obtained for an initial distribution along a straight line across the middle of the surface.

Case 5: specified strain rates are constant and isotropic throughout the canvas.. The specified strain rates are: $\mathbf{k}_{par} = \mathbf{k}_{per} = \mathbf{k}_{nor} = constant$. For a growth rate of k in every direction, the tissue should expand in every direction by a factor of e^{kt} in time t leaving no residual-strain (Figure 17A,B). That is, resultant-growth is the same as specified strain. There is no interaction between morphogens and none of them diffuse or dilute with growth.

Case 6: specified strain rates are constant and anisotropic throughout the canvas.. The tissue polarity is constant throughout the canvas. The specified strain rates are: $\mathbf{k}_{par} \neq \mathbf{k}_{per} \neq \mathbf{k}_{nor}$. As there are no growth conflicts, the resultant-growth should be the same as the specified strain and there will be no residual-strain. After a time t, the canvas grows by factors of $e^{\mathbf{k}_{par}t}$, $e^{\mathbf{k}_{per}t}$, and $e^{\mathbf{k}_{nor}t}$ in the principal directions (Figure 17C,D).

Case 7: specified strain rates are constant over time, anisotropic and vary along the uniform polarisation axis.. The tissue polarity is constant throughout the canvas (horizontal). The specified strain rates are: $\mathbf{k}_{par} = k.x$ (k is a constant), $\mathbf{k}_{per} = \mathbf{k}_{nor} = 0$. Under these conditions growth proceeds exactly as specified. In Figure 17E,F the growth rate in the initial canvas varies linearly across the canvas, from 0 at the left side to 1 at the right and polarisation is in the same direction. Fixing the left side at position $x = 0$, and setting the initial width to d, leads to a point which started at x moving over time t to

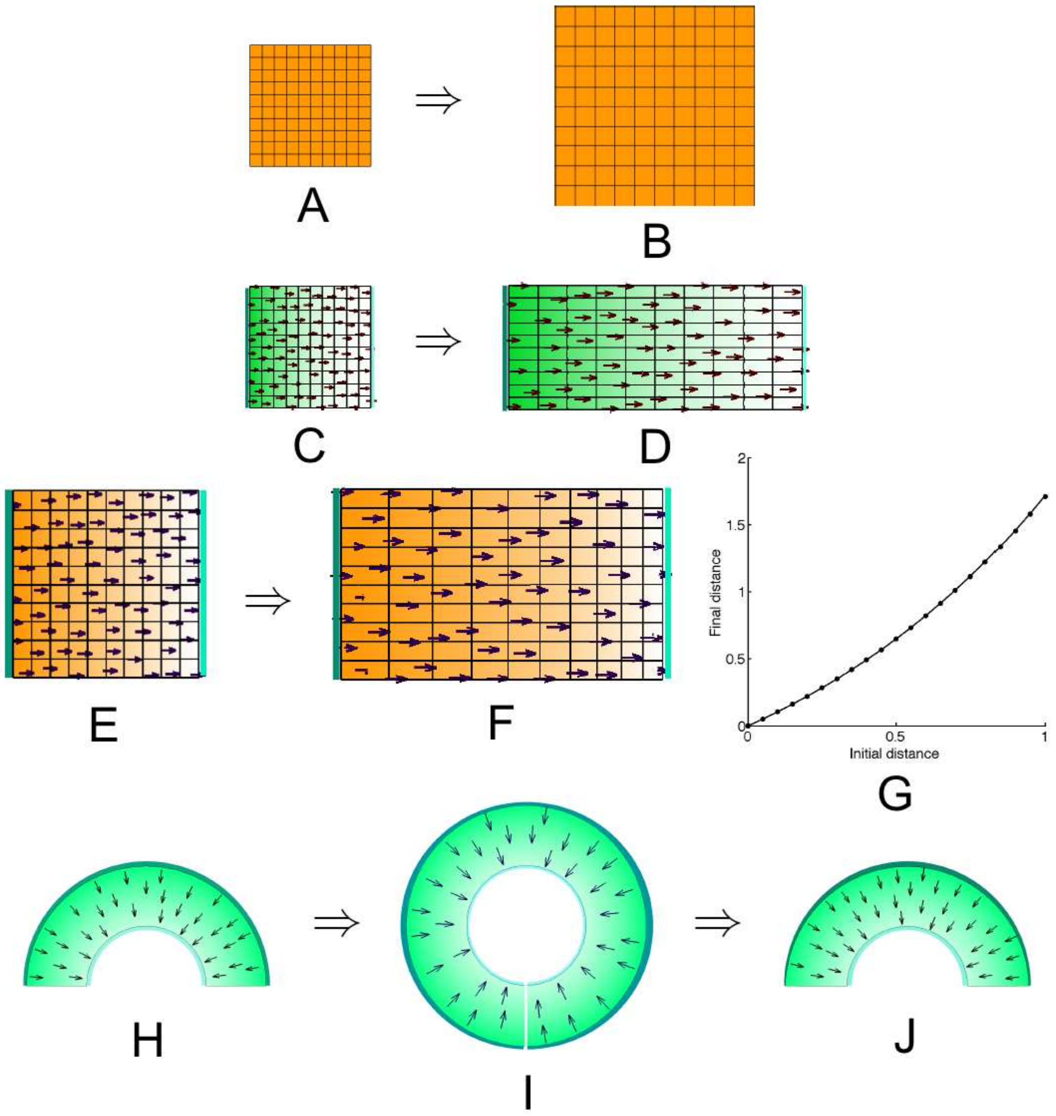

Figure 17. Cases 5 to 8, in which the resultant-growth is the same as specified strain. In Cases 5 to 7 the initial canvas is decorated with a square grid that deforms with growth. The grid is not the underlying mesh. Case 5: (A) Initial state $t = 0$, orange colour shows growth rate $\mathbf{k}_{par} = \mathbf{k}_{per} = \mathbf{k}_{nor} = 0.5$, $dt = 0.01$. (B) At $t = 1$. Case 6: (C) Initial state as for (A) but green shows polariser concentration (arrows show direction of gradient and the original organisers are in cyan), $\mathbf{k}_{par} = 1$ and $\mathbf{k}_{per} = 0$. The canvas now grows in only one direction, see (D) at $t = 1$. (E,F,G) Growth rate varies over the canvas. Case 7: (E) To compare computation with analysis; the polarisation (arrows) and growth profile (orange) before the start. $\mathbf{k}_{par}$ increases left to right and $\mathbf{k}_{per} = 0$. (F) After growing for 1 time unit note that the grid squares have deformed more on the right than the left. (G) The final positions of vertexes, as a function of their initial positions, relative to the left-hand edge. The theoretical result is the line and the computed results are dots. Case 8: (H,I,J) Showing that pure tangential growth leaves no residual, consistent with theory. (H) Level of polariser (green) and gradient (arrows). Growth rates are $\mathbf{k}_{per} > \mathbf{k}_{par} = 0$. (I) After growing for log(2) time units a ring is formed. (J) After negative growth for log(2) time units the shape exactly reverts.

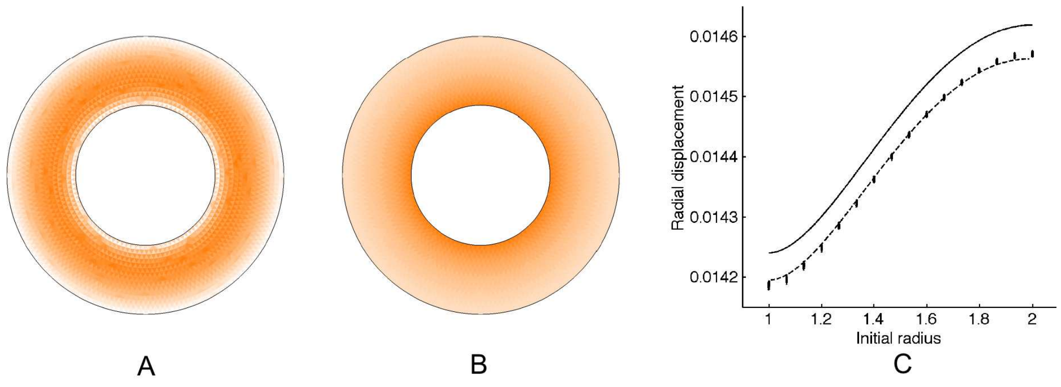

Figure 18. Case 9. Annulus growing in the tangential direction only. Poisson's ratio is zero. (A) Radial strain shown in orange. (B) Tangential strain shown in orange. (C) Radial displacement as a function of original radius, theoretical (solid line) and computed from the finite element model (dots).

$d(e^{xt} - 1)$. Figure 17G shows the agreement between this function (solid line) and the x coordinates of the vertex positions computed by *GFtbox* as a function of their original values (dots).

Case 8: Specified uniform anisotropic growth with non-uniform polarisation gradient. The tissue polarity is radial. The specified strain rates are: $\mathbf{k}_{per} > \mathbf{k}_{par} = 0$, $\mathbf{k}_{nor} = 0$. For a half-annulus constrained to the plane, uniform anisotropic growth in the tangential direction results in no residual-strain. There is uniform expansion of the canvas in that direction, while the inner and outer radii remain constant. Each canvas point travels along a circular arc about the centre. With a growth rate of 1, it will take time $\log 2 = 0.693$ to grow to a whole circle. Figure 17I shows the growth of such a half-annulus by *GFtbox* for $\log 2$ time units. An equal shrinking (growth rate $= -1$) for the same length of time should return the shape to its starting point, confirmed for *GFtbox* by Figure 17J.

Case 9: 2D Uniform anisotropic growth with non-uniform polarisation gradient. If a complete annulus constrained to the plane is caused to grow at a rate which is constant everywhere, and everywhere in the tangential direction, then in contrast to the half-annulus, residual strain will develop, as it cannot occupy more than 360 deg. It is possible to analytically compute the resulting deformation. (Compare [59], where the similar problem of a thick spherical shell is studied.)

The symmetry of the problem allows the deformation resulting from an infinitesimal amount of growth to be described by a function ζ mapping the original distance r of each point from the centre to its new distance $\zeta(r)$. For an arbitrary infinitesimal ζ, inner and outer radii r_0 and r_1, and an infinitesimal applied tangential growth field g, the radial strain is $d\zeta/dr - 1$ and the tangential strain is $\zeta/r - 1 - g$. For the case where Poisson's ratio is zero, the total strain energy described by the deformation is therefore (omitting some constant factors and writing ζ' for $\frac{d\zeta}{dr}$):

$$\int_{r_0}^{r_1} ((\zeta' - 1)^2 + (\zeta/r - 1 - g)^2)\, r\, dr$$

with the boundary conditions that $\zeta' = 1$ when r is equal to r_0 or r_1. Applying the calculus of variations we derive the following differential equation for the ζ that minimises the above integral:

$$\zeta - r\zeta' - r^2\zeta'' - gr = 0$$

This can be solved by Matlab's `ode45` solver given r_0, $\zeta'(r_0) = 1$, and $\zeta(r_0)$. A solution satisfying the boundary condition $\zeta'(r_1) = 1$ can be found by varying the unknown $\zeta(r_0)$. With $r_0 = 1$, $r_1 = 2$, and $g = 0.01$, and an annulus divided into 15 rings of finite elements, Figure 18 plots the radial displacement

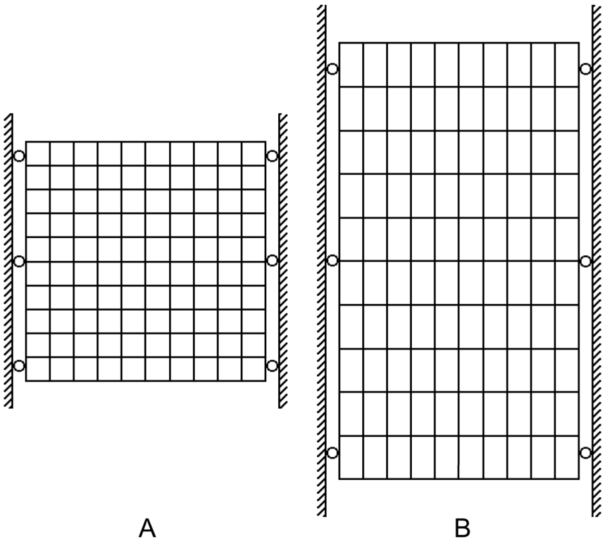

Figure 19. Case 10. Constrained growth. (A) A rectangle constrained between fixed frictionless walls. (B) The result of attempting to grow perpendicular to the walls is to instead grow parallel to them at a rate determined by Poisson's ratio.

$\zeta(r) - r$ against r for the finite element solution (points) and for two numerical solutions of the above equation (continuous and dashed lines). Each of the "points" is actually a set of between 95 and 180 points at equal initial distances from the centre. The solid line is the numerical solution for an annulus with $r_0 = 1$, $r_1 = 2$. The dashed line is the solution for $r_1 = 1.987$, a value chosen for the closest fit to the finite element solution. In other words, the finite element solution almost exactly coincides with the mathematical solution for an annulus with outer radius reduced by 1.3%. Note that the graph magnifies the apparent size of the error: the finite element model expands the inner edge from a radius of 1 to 1.01418 instead of 1.01424.

Case 10: 3D Effect of Poisson's ratio on growth. Consider a rectangle constrained at two opposite edges, by fixed frictioness walls, as shown in Figure 19. If the specified growth field attempts to make it grow horizontally by an amount g, then (assuming it does not buckle out of the plane) it will instead grow both vertically and in thickness by νg, where ν is Poisson's ratio. Computation in *GFtbox* produces this result. Taking $\nu = 0.3$ and a growth rate $\mathbf{k}_{par} = 1$, in one unit of time the mesh should grow vertically and in thickness by a factor of $e^{0.3} = 1.3499$. For a suitably chosen time step and tolerance, the simulation grows in height and thickness by 1.3441, an error of under 0.5%.

Case 11: 3D Uniform isotropic growth. In three dimensions, the example of uniform isotropic growth, Figure 20A,B.

Case 12: 3D Uniform anisotropic growth. Figure 20C,D.

Case 13: 3D Non-uniform anisotropic growth with uniform polariser gradient. Figure 20E,F.

Buckling edges

Case 14: Specified anisotropic growth is higher along one edge of the canvas. We include this example here because Sharon *et al.* [7] illustrate the effect of dissecting strips from the edge of a wavy-edged leaf and then flattening them, showing that the curvatures of the flattened strips increase the closer they are to the edge of the leaf. In our model, the gradient of polariser is uniform and aligned with the long axis of a rectangular canvas. The specified strain rates: $\mathbf{k}_{par} = k.x$ (k is a constant), $\mathbf{k}_{per} = \mathbf{k}_{nor} = 0$. The

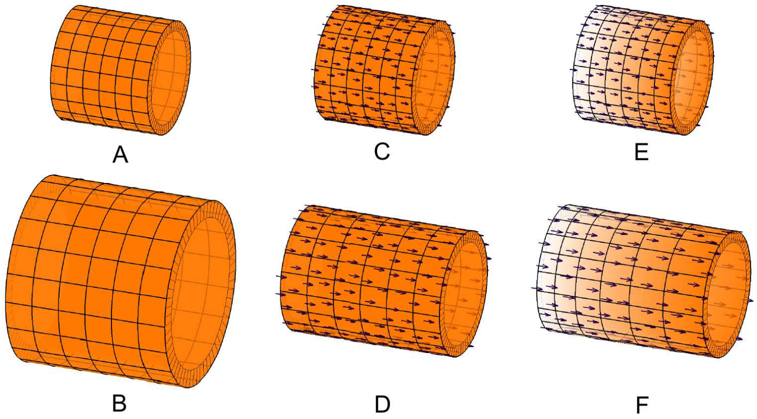

Figure 20. Cases 11 to 13. Three patterns of three-dimensional strain-free growth. Upper row: initial state, lower row: final state. (A,B) Uniform isotropic growth. (C,D) Uniform anisotropic growth. (E,F) Non-uniform anisotropic growth. Colour indicates longitudinal component of growth rate.

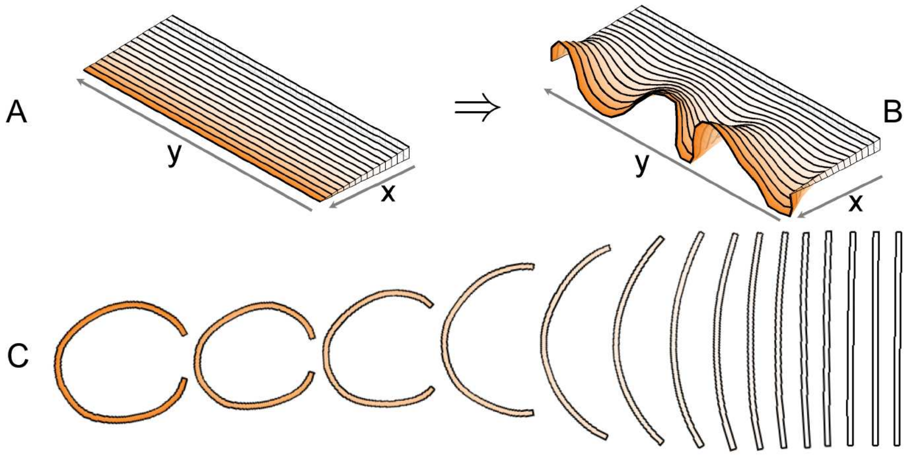

Figure 21. Case 14: Extra growth at the edge induces buckling. (A) The initial and final shape of a rectangular section from the edge of a canvas growing everywhere parallel to the edge, and more strongly nearer the edge, $\mathbf{k}_{par} = f(x)$, $\mathbf{k}_{per} = 0$. The tissue reduces in thickness (and hence stiffness) towards the edge. Colour indicates growth rate and black lines indicate where it will be dissected. The side and back edges are held fixed in order to simulate the presence of surrounding tissue. (B) The result of dissecting the grown canvas and computationally flattening the components. A movie of this development is in 'Video S8'.

initial canvas is thinner along the edge with high growth. This leads to buckling in waves and negative Gaussian curvature [51]. Depending on the way the growth rate and tissue stiffness vary with distance from the edge, a single wavelength of buckling may be favoured, or undulations that develop on multiple scales (c.f. [52]). *GFtbox* allows resultant shapes to be dissected and the components to be flattened onto a plane, while minimising distortion. Figure 21C illustrates the result of flattening similar strips cut from *GFtbox* model and the result of flattening the shape in Figure 21B. This result is similar to that observed in biological tissue [7].
